# Synthesis and structure of tricarbonyl(η^6^-arene)chromium complexes of phenyl and benzyl D-glycopyranosides

**DOI:** 10.3762/bjoc.8.118

**Published:** 2012-07-11

**Authors:** Thomas Ziegler, Ulrich Heber

**Affiliations:** 1 Institute of Organic Chemistry, University of Tuebingen, Auf der Morgenstelle 18, 72076 Tuebingen, Germany

**Keywords:** aryl glycosides, carbohydrates, transition-metal complex, tricarbonyl(arene)chromium

## Abstract

A series of 15 glycoside-derived tricarbonyl(η^6^-arene)chromium complexes were prepared in 19–87% yield by heating fully acetylated or methylated aryl O-, S-, N- and C-glycosides of D-glucopyranose and D-mannopyranose with hexacarbonylchromium. All tricarbonylchromium complexes were fully characterized. The structures of nine crystalline complexes were determined by X-ray diffraction, revealing unusual intra- and intermolecular nonclassical hydrogen bonds.

## Introduction

In 1957, Fischer and Öfele published the preparation of tricarbonyl(η^6^-benzene)chromium, which was the first arene tricarbonylchromium complex [1]. Since then, a plethora of transition-metal complexes of arenes have been prepared, characterized and described in the literature. Among the multitude of transition-metal complexes of aromatic compounds, however, only tricarbonyl(η^6^-arene)chromium compounds are widely used for organic syntheses [2–4]. This is due to the fact that tricarbonyl(η^6^-arene)chromium complexes are relatively stable compounds, which can be easily prepared and also easily reconverted into the parent arenes. Furthermore, the tricarbonylchromium group is an electron-withdrawing substituent increasing the acidity of the aromatic protons and the electrophilicity of the aromatic ring and, thus, making the arene more susceptible towards S_N_Ar reactions. Likewise, the benzylic and homo-benzylic positions in tricarbonyl(η^6^-arene)chromium complexes are more acidic and more prone to solvolysis, nucleophilic substitution and deprotonation than in the parent arenes due to the fact that the tricarbonylchromium ligand stabilizes both benzylic and homo-benzylic carbenium ions and carbanions [5–6]. Asymmetric ortho- or meta-substituted tricarbonyl(η^6^-arene)chromium compounds display planar chirality, which, in turn, makes these chiral complexes attractive catalysts for enantioselective reactions [6–8].

**Figure 1:**
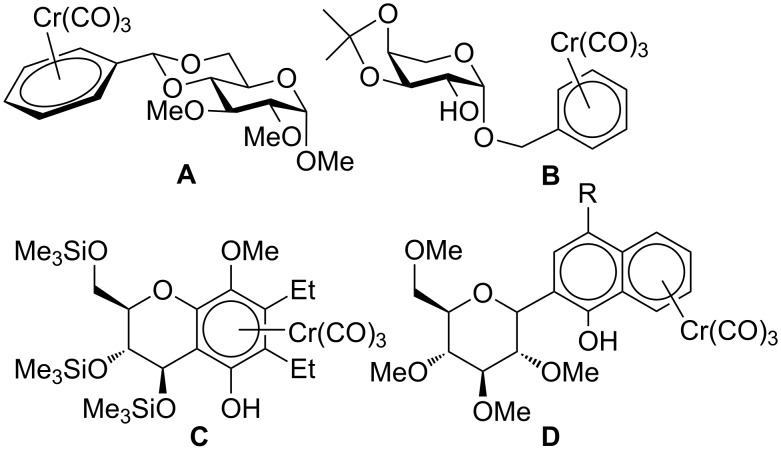
Known types of η^6^-tricarbonylchromium complexes of sugar derivatives [9–13].

Despite the broad applications that tricarbonyl(η^6^-arene)chromium complexes have found in organic synthesis since their discovery in 1957, only a very few tricarbonylchromium complexes of sugar derivatives are known today. Figure 1 shows the types of such carbohydrate-derived chromium complexes that have been described in the literature so far. Complexes of type **A** and **B** were obtained from the corresponding glycopyranosides and were studied as substrates for chiral-auxiliary-directed asymmetric ortholithiation and as catalysts for enantioselective Diels–Alder reactions [9–12]. Tricarbonylchromium complexes of type **C** and **D** were obtained via benzannulation of glucal-derived pentacarbonylchromium carbenes or by reaction of alkynyl C-glycosides with pentacarbonylchromium carbenes [13].

Further syntheses and characterizations of more examples of carbohydrate-derived tricarbonyl(η^6^-arene)chromium complexes are highly desirable in order to allow studies of the rich chemistry of such complexes in greater detail. In this paper we describe the synthesis and structural elucidation of a series of tricarbonyl-(η^6^-arene)chromium complexes of some simple phenyl and benzyl O-, N-, S- and C-glycosides.

## Results and Discussion

### Preparation of η^6^-tricarbonylchromium complexes of glycosides

In general, tricarbonyl(η^6^-arene)chromium compounds can be prepared in a wide variety of methods [4]. However, the following two methods are the most commonly employed ones: (a) ligand-exchange reaction between an arene and, most conveniently, either naphthalene–Cr(CO)_3_ complex or (MeCN)_3_Cr(CO)_3_ in which the chromium ligand is only weakly bound [14]; (b) simply heating the arene with hexacarbonylchromium in an inert solvent (Mahaffy–Pauson method) [15–16]. Method (a) has the disadvantage that the applied chromium complexes for the ligand exchange reaction are extremely sensitive toward oxidation, due to the weakly bound chromium. For method (b) high-boiling-point solvents, such as di-*n*-butylether, decalin or dioxane, can be used. The addition of THF to these solvents was shown to prevent the excessive sublimation of Cr(CO)_6_ during the formation of the arene–chromium complexes [17]. Therefore, we used method (b) for the preparation of tricarbonyl(η^6^-arene)chromium complexes of glycosides as follows.

One equivalent of phenyl or benzyl glycoside **1** and one equivalent of Cr(CO)_6_ were dissolved in di-*n*-butylether containing 10% THF, and the solution was stirred at 140 °C under argon and under the exclusion of light (brown glassware). After the reaction of **1** with Cr(CO)_6_ was complete (16–96 h), the solvent was evaporated and the crude complexes **2** were purified by chromatography under argon on silica gel with *n*-hexane/ethyl acetate mixtures as eluent. Crystalline chromium complexes **2** were recrystallized from ethanol, and for suitable crystals X-ray structures were determined. Table 1 summarizes the results for the preparation of the complexes **2** from simple acetylated and methylated phenyl, benzyl and 1-*O*-benzoyl glycosides **1**. All glycosides **1a**–**k** were prepared according to literature procedures (for details see the Supporting Information File 1).

**Table 1:** Synthesis of η^6^-tricarbonylchromium complexes **2a**–**k** from glycosides **1a**–**k** and Cr(CO)_6_ in di-*n*-butylether/THF 9:1 at 140 °C under Ar and exclusion of light.

Entry	Glycoside **1**	Time	Complex **2**	Yield

1	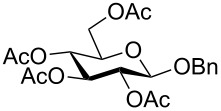 **1a**	96 h	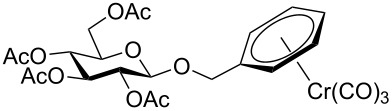 **2a**	29%
2	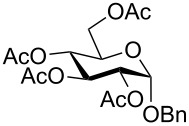 **1b**	90 h	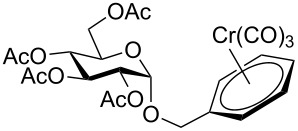 **2b**	87%
3	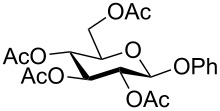 **1c**	80 h	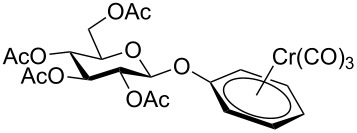 **2c**	29%
4	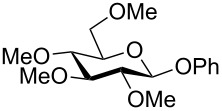 **1d**	16 h	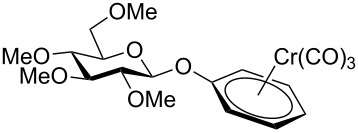 **2d**	19%
5	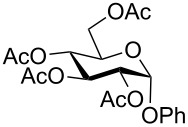 **1e**	70 h	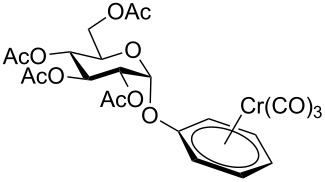 **2e**	53%
6	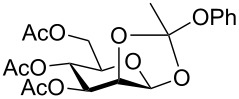 **1f**	42 h	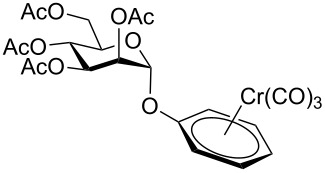 **2f**	47%
7	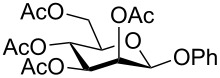 **1g**	42 h	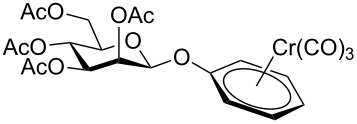 **2g**	35%
8	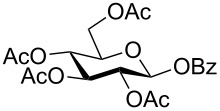 **1h**	67 h	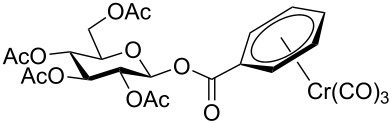 **2h**	30%
9^a^	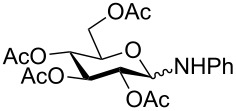 **1i**	24 h	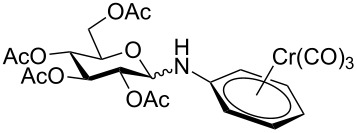 **2i**	46%
10	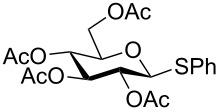 **1j**	24 h	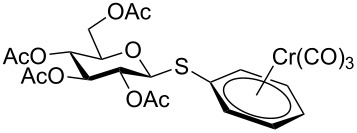 **2j**	49%
11	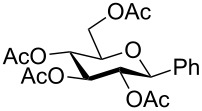 **1k**	80 h	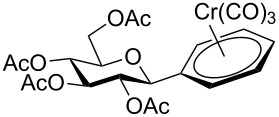 **2k**	81%

^a^Anomeric mixture α:β = 1:2.

All reactions of glycosides **1a**–**k** with Cr(CO)_6_ proceeded smoothly and gave the corresponding tricarbonyl(η^6^-arene)chromium complexes **2a**–**k** in medium yield. The somewhat lower yields in some cases are due to oxidative decomposition of the products during purification by column chromatography on silica gel.

The reaction times also varied significantly between 16 and 96 h. This was due to the purity of the hexacarbonyl chromium charges we purchased from several companies. In general, however, electron-donating protecting groups in the sugar moiety accelerated the formation of the chromium complexes at the aglycon (Table 1, entries 3 and 4). Treatment of the mannose orthoester **1f** (Table 1, entry 6) under the standard conditions applied here resulted in the exclusive formation of tricarbonyl[(2,3,4,6-tetra-*O*-acetyl-α-D-mannopyranosyloxy)-η^6^-benzene]chromium (**2f**) in 47% yield. In the glucose series it is well known that orthoesters similar to **1f** rearrange to the corresponding glycosides upon heating [18]. Therefore, it is very likely that **1f** isomerized to the corresponding phenyl 2,3,4,6-tetra-*O*-acetyl-α-D-mannopyranoside, which was then converted into complex **2f**. The direct complexation of orthoester **1f** is unlikely because no such chromium complex was detected. The anomeric α-configuration of **2f** was proven by decomplexation of an analytical sample with iodine in CHCl_3_ followed by measurement of the NMR spectrum of the formed glycoside. The latter showed a CH-coupling constant at the anomeric center of 173.9 Hz, which is indicative of an α-anomer [19]. As additional proof for the anomeric configuration of **2f**, we also prepared β-anomer **2g** (Table 1, entry 7). For the preparation of chromium complex **2i** we used a 1:2 anomeric mixture of aminoglucoside **1i**, which did not change during the complexation (Table 1, entry 9).

**Scheme 1:**
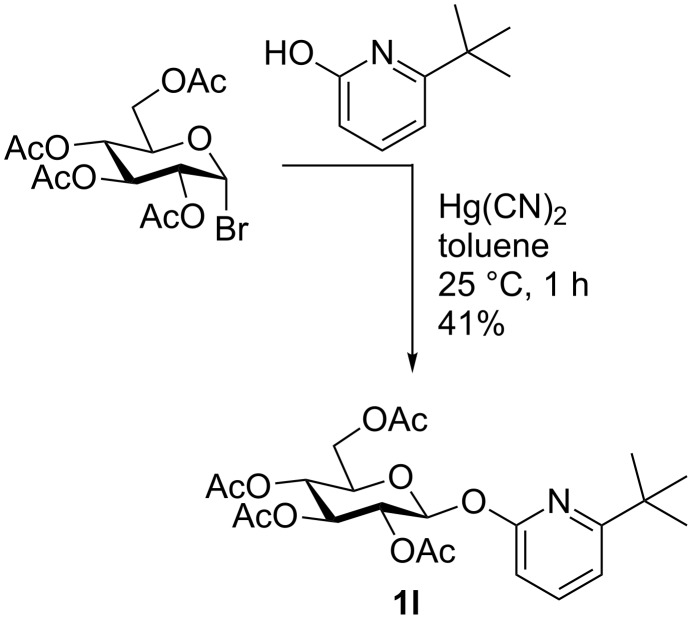
Synthesis of glucoside **1l**.

For the anticipated synthesis of a glucose-derived tricarbonyl(η^6^-pyridine)chromium complex we prepared glycoside **1l** by reacting acetobromoglucose with 6-*tert*-butyl-2-hydroxypyridine [20] under Helferich conditions (Hg(CN)_2_). Only the corresponding *O*-glycoside **1l** was obtained, and no *N*-glycoside was formed in this Helferich glycosylation (Scheme 1). Sterically hindered 6-*tert*-butyl-2-hydroxypyridine was chosen as the aglycon in order to avoid complexation of hexacarbonylchromium with the basic nitrogen atom. However, all attempts to convert **1l** into the corresponding tricarbonyl(η^6^-pyridine)chromium complex failed. Attempts to prepare a chromium complex of **1l** by a ligand exchange reaction with naphthalene–Cr(CO)_3_ or (MeCN)_3_Cr(CO)_3_ were also unsuccessful (no further experimental details shown).

Next, we also prepared some sugar-derived tricarbonylchromium complexes of glycosides having a prochiral aglycon, i.e., an ortho-substituted phenyl or benzyl aglycon. Glycosides **1m** [21] and **1q** [22] were prepared according to the respective literature procedures. Glycosides **1n**–**p** were not known in the literature and were prepared as follows: *o*-Tolyl 2,3,4-6-tetra-*O*-acetyl-β-D-glucopyranoside (**1m**) was first deacetylated (cat. NaOMe in MeOH) followed by treatment with pivaloyl chloride in pyridine to give **1n** in 86% yield. Treatment of pentaacetylglucose and 2-*tert*-butylphenol with BF_3_-etherate in dichloromethane afforded **1o** in 31% yield. Glycoside **1p** was prepared by a Helferich glycosylation of *o*-methylbenzyl alcohol with acetobromoglucose. For further details see Supporting Information File 1. Table 2 summarizes the results of the complexation of glucosides **1m**–**q** affording the diastereomeric tricarbonyl(η^6^-arene)chromium complexes **2m**–**q**.

**Table 2:** Synthesis of tricarbonylchromium complexes **2m**–**q** from glycosides **1m**–**q** containing a prochiral aglycon and Cr(CO)_6_ in di-*n*-butylether/THF 9:1 at 140 °C under Ar in the dark.

Entry	Glycoside **1**	Time	Complex **2**	Yield	Ratio^a^

1	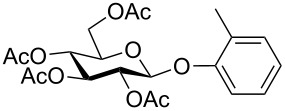 **1m**	70 h	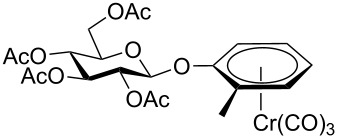 ***pR*-2m** 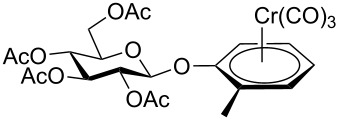 ***pS*-2m**	76%	***pR*-2m**:***pS*-2m** = 7:3^b^
2	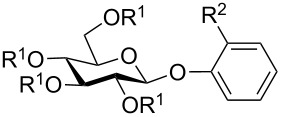 **1n** R^1^ = Piv, R^2^ = Me **1o** R^1^ = Ac, R^2^ = C(CH_3_)_3_		no product	–	–
3	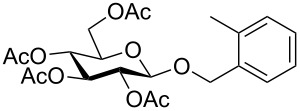 **1p**	15 h	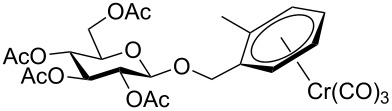 **2p**	42%	***pR*-2p**:***pS*-2p** = 1:1^c^
4	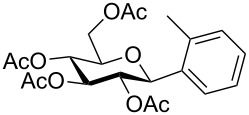 **1q**	16 h	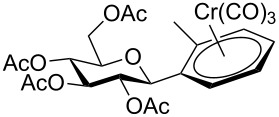 **2q**	42%	***pR*-2q**:***pS*-2q** = 1:1^c^

^a^Determined by ^1^H NMR; ^b^diastereomers can be separated by crystallization; ^c^no separation possible.

Treatment of *o*-tolyl glucoside **1m** with hexacarbonylchromium under the standard conditions for 70 h afforded a 7:3 mixture of the diastereomeric tricarbonylchromium complexes **2m** in 76% overall yield (Table 2, entry 1). Upon slow crystallization of the diastereomeric mixture from ethanol, isomer ***pR*-2m** could be obtained in pure form. From the mother liquor a small amount of pure isomer ***pS*-2m** could be isolated upon repeated recrystallization as well. The absolute configuration of the planar chiral tricarbonyl(η^6^-*o*-tolyl)chromium aglycon in both diastereomers **2m** could be unambiguously assigned by X-ray crystallography. Since the two diastereomers **2m** were not formed in equal amounts during complexation of glucoside **1m**, we contemplated that an even higher diastereoselectivity should be obtained when sterically more demanding aglycons or sugar moieties are present during reaction with Cr(CO)_6_. Therefore, we also reacted glucosides **1n** and **1o** bearing either bulky pivaloyl groups or a *tert*-butyl group in the aglycon in the sugar moiety with Cr(CO)_6_. However, no complexation could be detected even under prolonged reaction time (Table 2, entry 2). Placing the *o*-tolyl group in a position more distant from the sugar part, as in glucoside **1p** (Table 2, entry 3), or using *o*-tolyl C-glucoside **1q** (Table 2, entry 4) gave 1:1 mixtures of the corresponding diastereomers **2p** and **2q**, respectively. A separation of the diastereomers by crystallization was not possible for complexes **2p** and **2q**.

**Scheme 2:**
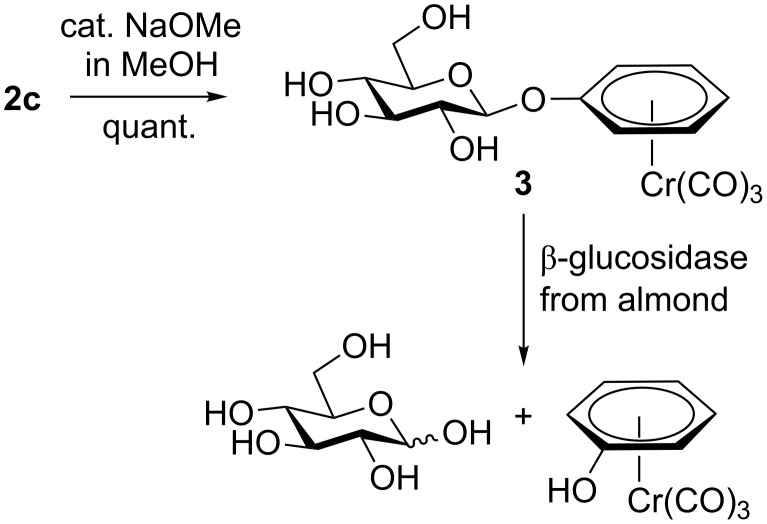
Deprotection of **2c** and enzymatic cleavage of **3**.

The *O*-acetylated carbohydrate-derived tricarbonyl(η^6^-arene)chromium complexes prepared here can be deprotected without affecting the chromium complex, as exemplified in Scheme 2. Zemplén deacetylation of **2c** afforded tricarbonyl(β-D-glucopyranosyloxy-η^6^-benzene)chromium (**3**) in quantitative yield. Glucoside **3** is a good substrate for β-glucosidases, such as almond glucosidase E.C. 3.2.1.21, and is converted into D-glucose and tricarbonyl(η^6^-phenol)chromium in 98% yield.

### Structures of tricarbonyl(η^6^-arene)chromium complexes of glycosides

In general, complexation of glycosides **1** with Cr(CO)_6_ to give the corresponding tricarbonyl(η^6^-arene)chromium compounds **2** does not significantly affect the conformation of the sugar moieties. This is evident from the ^1^H NMR spectra of compounds **2**, which show no significant change of chemical shifts and coupling constants of the carbohydrate protons compared to those of the educts **1**. As an example, Table 3 shows the chemical shifts of the protons of compounds **1a** and **2a**. All NMR signals of the sugar protons remain practically unchanged, whereas the protons of the benzylic methylene group and the benzene moiety appear, as was expected, at higher fields in **2a**. Therefore, it can be concluded that the sugar rings remain in the ^4^*C*_1_ conformation. The high-field shifts of the benzylic methylene group and the aromatic moiety are in accordance with other tricarbonyl(η^6^-arene)chromium complexes. This is further confirmed by the X-ray structures obtained from crystalline chromium complexes.

**Table 3:** Chemical shifts (δ in ppm) of the protons of compounds **1a** and **2a** derived from the ^1^H NMR spectra of the compounds measured in acetone-*d*_6_.

	H1	H2	H3	H4	H5	H6a	H6b	OCH_2_	H_Ar_

**1a**	4.83 d	4.98 t	5.25 t	5.05 t	3.97 m	4.29 dd	4.15 dd	4.87 d 4.67 d	7.28–7.34 m
**2a**	4.98 d	4.98 t	5.28 t	5.06 t	4.01 m	4.27 dd	4.16 dd	4.68 d 4.45 d	5.56–5.72 m

For the tricarbonylchromium complexes **2a**–**e**,**j**,**k**,**m** which gave suitable crystals, structures were determined by X-ray diffraction [23]. Crystals were grown for all compounds by slow crystallization of the compounds from ethanol. The conformation of the Cr(CO)_3_ group in relation to the aromatic ring shows some deviations compared to other tricarbonyl(η^6^-arene)chromium complexes. In complexes where the benzene ring carries an electron-donating substituent, the Cr(CO)_3_ group is preferably in an eclipsed orientation, whereas in cases where the benzene carries an electron-withdrawing substituent, a staggered conformation is commonly found [24]. Here, eclipsed conformations of the Cr(CO)_3_ group were found in compounds **2c** and **2e**, which both carry an electron-donating glycosyloxy substituent. However, compound **2k**, which carries an electron-withdrawing substituent also shows an eclipsed conformation of the Cr(CO)_3_ group. Likewise, all compounds **2a**, **2b**, **2d**, **2j** and **2m** show a staggered conformation of the Cr(CO)_3_ group, although they all carry an electron-donating substituent at the benzene ring. All complexes show unusual intra- and intermolecular nonclassical hydrogen bonds [25], which will be discussed in more details for each individual X-ray structure in the following. For technical details of the X-ray structures see Supporting Information File 1.

**Figure 2:**
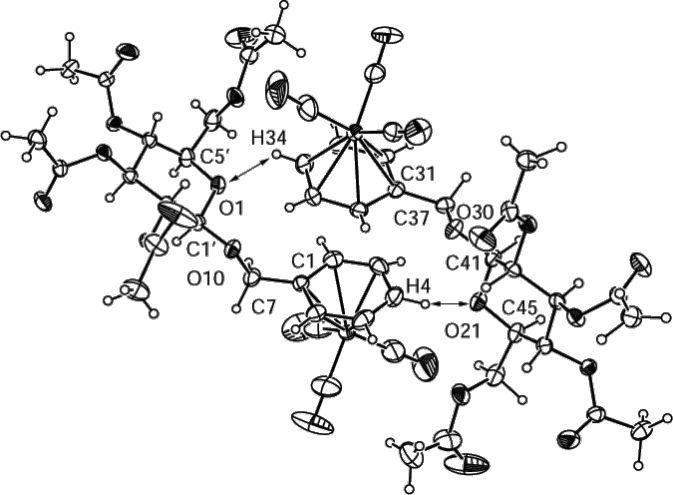
ORTEP-plot of the asymmetric unit containing two molecules of compound **2a** showing 30% probability ellipsoids; the arrows show the nonclassical H-bonds between O1 and H34 and O21 and H4.

Figure 2 shows the asymmetric unit of compound **2a**, which contains two slightly differently distorted molecules. Table 4 summarizes some selected atomic distances and angles of the two molecules in the asymmetric unit (**2aa** refers to the right molecule and **2ab** to the left one in Figure 2). Most significantly, the biggest differences are found around the benzylic methylene groups (C7 and C37, respectively). Another significant feature of **2a** that was not observed in any other complex **2** is the parallel face-to-face orientation of the two benzene rings. However, the distance of the two ring planes of 3.627 Å and the angle of 16° between the ring planes indicate no π-interaction of the two benzene rings.

**Table 4:** Selected corresponding distances and angles of **2aa** and **2ab**.

**2aa**		**2ab**	

C1’–O10	1.375 Å	C41–O30	1.378 Å
O10–C7	1.427 Å	O30–C37	1.407 Å
C7–C1	1.491 Å	C37–C31	1.477 Å
C1’–O10–C7	112.8°	C41–O30–C37	113.2°
C1–C7–O10	108.4°	C31–C37–O30	112.6°
C1’–O10–C7–C1	147.8°	C41–O30–C37–C31	157.9°

The most significant features in the crystal structure of **2a** are the two intermolecular nonclassical hydrogen bonds between O1 and H34 and O21 and H4 (Figure 2). That these hydrogen bonds are indeed true bonds is evident from the distances of 2.659 Å for O1/H34 and 2.680 Å for O21/H4 and the angles 101.9° for C1’–O1–H34, 123.7° for C5’–O1–H34, 122.8° for C41–O21–H4 and 105.6° for C41–O21–H4. These distances and angles allow protons H4 and H34 to interact with the lone pairs of the oxygen atoms O1 and O21 of the sugar rings [26]. Most likely, the two nonclassical hydrogen bonds in **2a** are the reasons why two molecules crystallize as slightly different pairs; a feature not observed in the other complexes.

**Figure 3:**
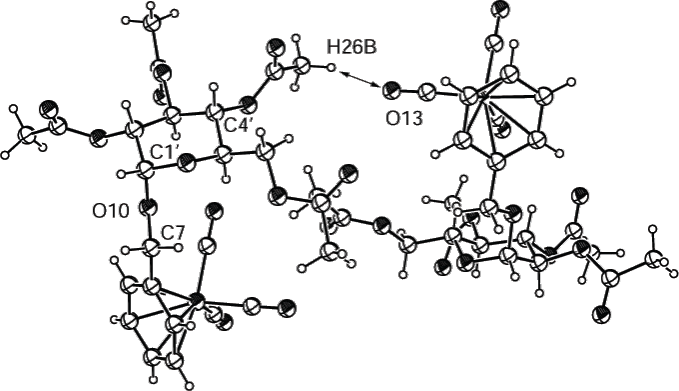
ORTEP-plot of the asymmetric unit showing two molecules of compound **2b** and 30% probability ellipsoids; the arrows show the nonclassical H-bonds between O13 and H26B.

Figure 3 shows the structure of compound **2b**, which is the anomer of **2a**. Once again a nonclassical hydrogen bond is responsible for the interaction of the molecules. Contrary to **2a**, in which a nonclassical H-bond was found between the oxygen of a sugar ring and the hydrogen of a benzene ring, the H-bond forms in **2b** between the oxygen of a carbonyl group (O13) and the hydrogen of an acetyl group (H26B). However, the length of this H-bond (2.212 Å) and the angles (161.6° for C≡O···H and 159.8° for C–H···O) prove the presence of the H-bond.

**Figure 4:**
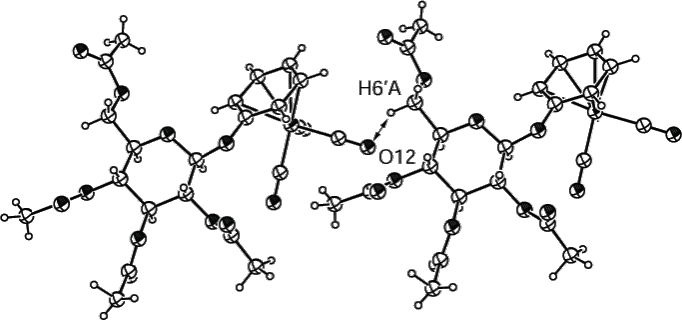
ORTEP-plot of the asymmetrical unit showing two molecules of compound **2c** and 30% probability ellipsoids; the arrows show the nonclassical H-bonds between O12 and H6’A.

Figure 4 shows the structure of compound **2c**. Once again the most significant feature is a nonclassical intermolecular hydrogen bond between the oxygen of a carbonyl group (O12) and the hydrogen (H6’A) at C6 of the neighboring molecule. Distance (2.628 Å) and C≡O···H and C–H···O angles (123 and 101.5°) are indicative of the H-bond. Compound **2c** also shows that complexation of an aromatic aglycon with tricarbonylchromium does not have any significant influence on the sugar ring. In Table 5, the distances and angles at the anomeric center of **2c** are compared to those for phenyl β-D-glucopyranoside (Ph-Glc) [27] showing that only the anomeric bond is slightly shortened upon complexation.

**Table 5:** Comparison of selected distances and angles in **2c** with phenyl β-D-glucopyranoside.

	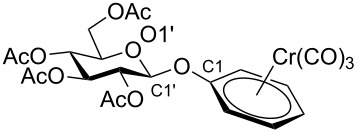	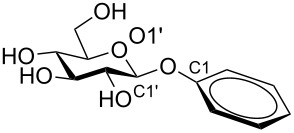

Bond	**2c**	phenyl β-D-glucopyranoside

C1’–O1	1.417 Å	1.394 Å
O1’–C1’	1.418 Å	1.434 Å
O1–C1	1.360 Å	1.388 Å
C1’–O1–C1	119.1°	118.0°
O1’–C1’–O1	106.7°	107.4°

**Figure 5:**
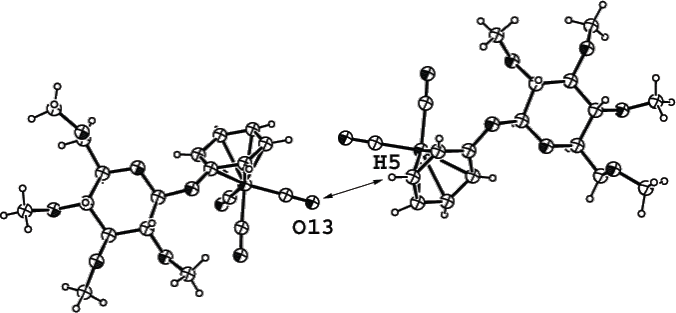
ORTEP-plot of the asymmetric unit showing two molecules of compound **2d** and 30% probability ellipsoids; the arrows show the nonclassical H-bonds between O13 and H5.

Figure 5 shows the structure of compound **2d**, which contains a hydrogen bond between carbonyl oxygen O13 of the left molecule and hydrogen H5 of the benzene ring of the right molecule. The length of this hydrogen bond is 2.498 Å and the angles C≡O···H and C–H···O are 166.2 and 158.3°, respectively. All atomic distances and angles of the tricarbonyl(η^6^-benzene)chromium aglycon are almost identical to those of the acetylated counterpart **2c**. However, in **2d**, the carbonyl groups are in a staggered conformation (like in compound **2a**) whereas in **2c** the carbonyl ligands adopt an eclipsed conformation.

**Figure 6:**
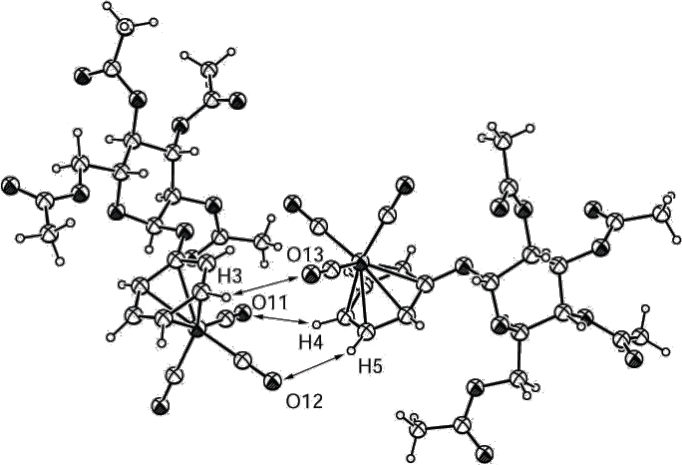
ORTEP-plot of the asymmetrical unit showing two molecules of compound **2e** and 30% probability ellipsoids; the arrows show the nonclassical H-bonds between O13 and H3, O11 and H4 and O12 and H5.

All bond distances and angles for both the carbohydrate moiety and the tricarbonyl(η^6^-benzene)chromium aglycon in compound **2e** (Figure 6) are within the values found for all of the other complexes. The carbonyl groups adopt an eclipsed conformation like in **2c**. In the crystal, two complexed benzene rings face each other and are aligned parallel, with the tricarbonylchromium groups facing in opposite directions. One carbonyl group of each molecule is placed in between the benzene rings and, thus, allows for the formation of a “network” of three nonclassical H-bonds in the crystal. The lengths and angles of these three hydrogen bonds in **2e** are within the typical range for nonclassical H-bonds [28–31] (Table 6).

**Table 6:** Bond lengths and angles for the nonclassical H-bonds in **2e**.

H-bond	Length C≡O···H	Angle C–H···O	Angle C≡O···H

O11–H4	2.594 Å	154.4°	94.5°
O12–H5	2.668 Å	142.5°	118.9°
O13–H3	2.656 Å	133.4°	125.6°

**Figure 7:**
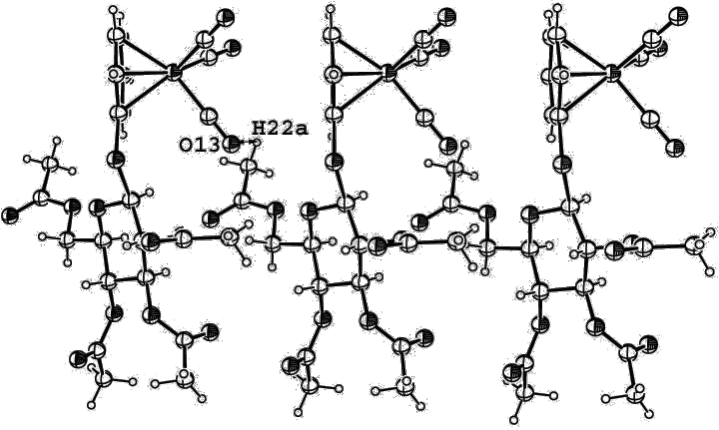
ORTEP-plot of the asymmetric unit showing three molecules of compound **2j** and 30% probability ellipsoids; the arrows show the nonclassical H-bonds between O13 and H22a.

Figure 7 shows the crystal structure of the *S*-glycoside **2j** in which the molecules are stacked with all benzene rings in an almost symmetric parallel orientation on one side. However, an interaction of the tricarbonyl(η^6^-benzene)chromium rings can be excluded because the distance between the benzene rings is, with >3.37 Å, too long for such interactions, which only occur at shorter distances [32–34]. A nonclassical hydrogen bond is found between O13 of a carbonyl group and H22a of the acetyl group at O6 of the sugar moiety. The bond lengths and angles of this H-bond are in the expected range (2.709 Å for O···H, 138.2° for angle C–H···O and 93.5° for angle C≡O···H).

**Figure 8:**
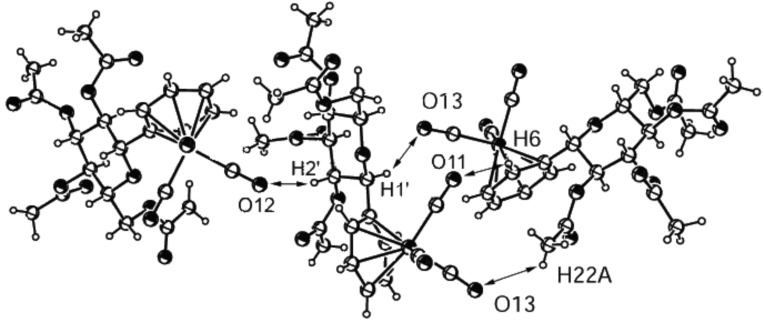
ORTEP-plot of the asymmetric unit showing three molecules of compound **2k** and 30% probability ellipsoids; the arrows show the nonclassical H-bonds between O12 and H2’, O13 and H1’, O11 and H6 and O13 and H22A.

The structure of the C-glycoside **2k** (Figure 8) also shows a “network” or nonclassical hydrogen bond in the crystal. The molecules group similarly to compound **2e**; however, the benzene rings between two molecules (Figure 8, molecules on the right side) are not oriented in parallel. Four nonclassical hydrogen bonds form between the oxygen atoms of three carbonyl groups (O12 and O13) and the hydrogen atoms of two methyl groups (H2’ and H22a), one methylene group (H1’) and one aromatic hydrogen (H6). Table 7 summarizes the bond lengths and angles of these nonclassical H-bonds.

**Table 7:** Bond lengths and angles for the nonclassical H-bonds in **2k**.

H-bond	Length C≡O···H	Angle C–H···O	Angle C≡O···H

O11–H6	2.714 Å	129.5°	126.3°
O13–H22a	2.719 Å	127.1°	118.9°
O13–H1’	2.720 Å	150.5°	118.4°
O12–H2’	2.632 Å	164.2°	130.3°

**Figure 9:**
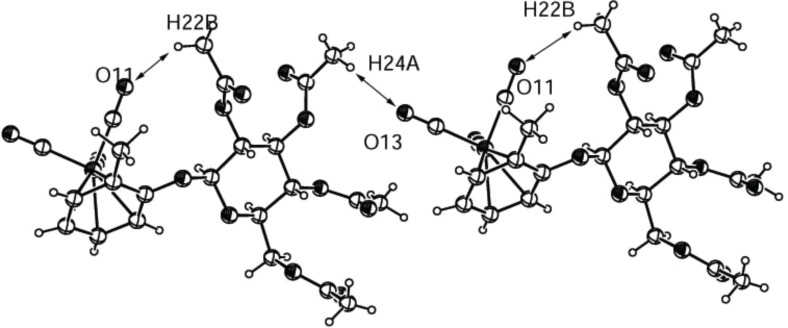
ORTEP-plot of the asymmetric unit showing two molecules of compound ***pR*-2m** and 30% probability ellipsoids; the arrows show the nonclassical intramolecular H-bonds between O11 and H22B and the intermolecular ones between O13 and H24A.

**Figure 10:**
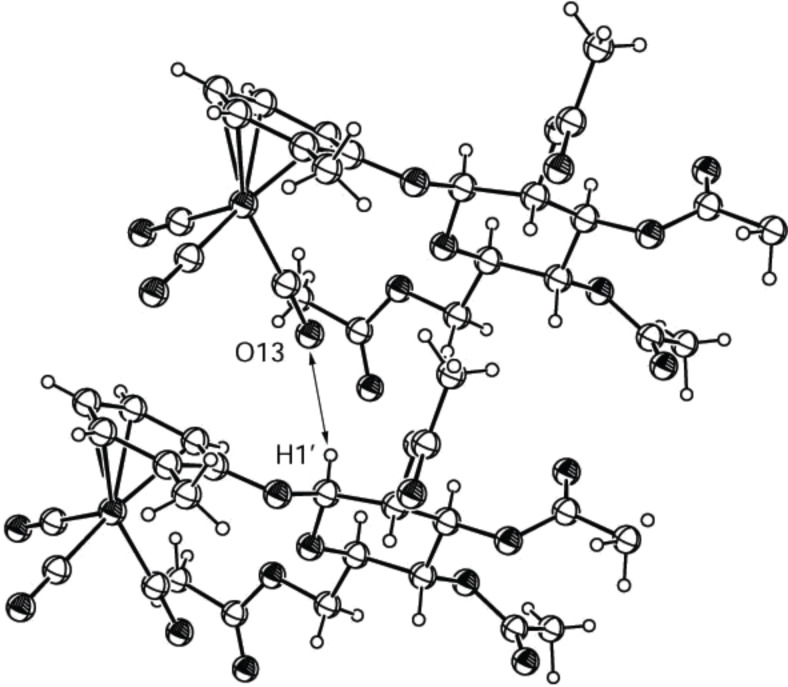
ORTEP-plot of the asymmetric unit showing three molecules of compound ***pS*-2m** and 30% probability ellipsoids; the arrows show the nonclassical H-bonds between O13 and H1’.

Figure 9 and Figure 10 show the crystal structures of the two diastereomers **2m**. The isomer with (*pR*)-configuration of the tricarbonyl(η^6^-benzene)chromium group (Figure 9) shows one intramolecular nonclassical hydrogen bond between carbonyl oxygen O11 and H22B of the 2-*O*-acetyl group and one intermolecular H-bond between carbonyl oxygen O13 of the right molecule and H24A of the 3-*O*-acetyl group of the left molecule. All bond lengths and angles of these hydrogen bonds are in the expected range for nonclassical H-bonds.

The structure of the diastereomer **2m** with (*pS*)-configuration of the tricarbonyl(η^6^-benzene)chromium group (Figure 10) resembles compound **2j** (Figure 7) in that the benzene rings are also in a symmetric parallel orientation. However, the distance of the benzene rings is, with 3.178 Å, shorter than in **2j** but still too long for an interaction of the aromatic rings. One nonclassical hydrogen bond is found in ***pS*-2m,** between the oxygen O13 of a carbonyl group and the anomeric hydrogen H1’ of the sugar moiety. The bond lengths and angles of this H-bond are also in the expected range for nonclassical hydrogen bonds (2.699 Å for O···H, 128.7 degree for angle C–H···O and 167.3 degree for angle C≡O···H).

## Conclusion

We have prepared and characterized a series of tricarbonyl(η^6^-benzene)chromium complexes from phenyl and benzyl O-, N-, S- and C-glycosides, and which were hitherto unknown. The X-ray diffraction of some of these glycoside-derived tricarbonylchromium complexes revealed crystal structures that contain numerous nonclassical hydrogen bonds.

## Experimental

**General details.** All solvents were dried and distilled prior to their use. Reactions were performed under Ar and monitored by TLC on Polygram Sil G/UV silica gel plates from Machery & Nagel. Detection was effected by charring with H_2_SO_4_ (5% in EtOH) or by inspection of the TLC plates under UV light. Reactions involving Cr(CO)_6_ or chromium complexes were performed in brown glassware or in the dark. NMR spectra were recorded on a Bruker ARX 250 spectrometer at 250 MHz for proton spectra and 62.5 MHz for carbon spectra, on a Bruker Avance 400 spectrometer at 400 MHz for proton spectra and 100 MHz for carbon spectra, and on a Bruker AMX 600 spectrometer at 600 MHz for proton spectra and 150 MHz for carbon spectra. Tetramethylsilane was used at the internal standard. Chemical shifts δ are given in parts per million (ppm) and coupling constants in hertz (Hz). All NMR spectra were treated as first-order spectra. HRMS was performed on a Bruker Daltonics APEX 2 FT–ICR spectrometer. FAB–MS was performed on a Finnigan MAT TSQ 70 spectrometer and ionization with Xe. IR spectra were recorded with a Bruker Tensor 27 IR spectrometer. UV spectra were recorded with a Shimadzu UV 2102 PC spectrometer. Elemental analyses were performed on a Hekatech Euro 3000 CHN analyzer. Optical rotations were measured with a Perkin-Elmer Polarimeter 341. Melting points were determined with a Büchi B-540 apparatus and are uncorrected. Preparative chromatography was performed on silica gel (0.032–0.063 mm) from Machery & Nagel with different mixtures of solvents as eluent.

**Starting materials.** The following glycosides **1** were prepared according to literature procedures: Benzyl 2,3,4,6-tetra-*O*-acetyl-β-D-glucopyranoside (**1a**) [35], benzyl 2,3,4,6-tetra-*O*-acetyl-α-D-glucopyranoside (**1b**) [36], phenyl 2,3,4,6-tetra-*O*-acetyl-β-D-glucopyranoside (**1c**) [37], phenyl 2,3,4,6-tetra-*O*-methyl-β-D-glucopyranoside (**1d**) [38], phenyl 2,3,4,6-tetra-*O*-acetyl-α-D-glucopyranoside (**1e**) [39], 3,4,6-tri-O-acetyl-1,2-(1-phenoxy-1-ethylidene)-β-D-mannopyranose (**1f**) [37], phenyl 2,3,4,6-tetra-*O*-acetyl-β-D-mannopyranoside (**1g**) [40], benzoyl 2,3,4,6-tetra-*O*-acetyl-β-D-glucopyranoside (**1h**) [41], *N*-phenyl-2,3,4,6-tetra-*O*-acetyl-D-glucopyranosylamine (**1i**) [42], phenyl 2,3,4,6-tetra-*O*-acetyl-1-thio-β-D-glucopyranoside (**1j**) [43], 2,3,4,6-tetra-*O*-acetyl-β-D-glucopyranosylbenzene (**1k**) [44], 2-methylphenyl 2,3,4,6-tetra-*O*-acetyl-β-D-glucopyranoside (**1m**) [21], 2-(2,3,4,6-tetra-*O*-acetyl-β-D-glucopyranosyl)methylbenzene (**1q**) [22].

**Chromium complexes: general procedure.** A solution of glycoside **1** (1 mol equiv) and Cr(CO)_6_ (1 mol equiv) in di-*n*-butylether/THF 9:1 was heated in the dark under Ar at 140 °C until TLC indicated complete consumption of **1** and was then concentrated. Chromatography of the residue under Ar with *n*-hexane/ethyl acetate 2:1 and immediate concentration of the fractions containing the chromium complex gave **2**. Crystalline complexes **2** were slowly recrystallized from ethanol. Suitable crystals were submitted to X-ray crystallographic analysis.

### Tricarbonyl[(2,3,4,6-tetra-*O*-acetyl-β-D-glucopyranosyloxymethyl)-η^6^-benzene]chromium (**2a**)

Treatment of **1a** (3.0 g, 6.8 mmol) and Cr(CO)_6_ (1.50 g, 6.8 mmol) in di-*n*-butylether/THF (100 mL) for 96 h according to the general procedure afforded **2a** (1.14 g, 29%) as yellow triclinic crystals: Mp 140–141 °C (EtOH); [α]_D_ −11.0 (*c* 1.0, toluene); IR (KBr): 1952, 1895 cm^−1^; FAB–MS (*m*/*z*): 597 [M + Na]^+^, 574 [M]^+^, 490 [M − 3CO]^+^; ^1^H NMR (acetone-*d*_6_) δ 5.74–5.56 (m, 5H, H-aryl), 5.28 (t, 1H, 3-H), 5.06 (t, 1H, 4-H), 4.98 (t, 1H, 2-H), 4.98 (dd, *J*_1,2_ = 7.3 Hz, 1H, 1-H), 4.68, 4.45 (dd, 2H, OCH_2_Ph), 4.27 (dd, 1H, 6a-H), 4.16 (dd, 1H, 6b-H), 4.01 (m, 1H, 5-H), 2.06–1.94 (m, 12H, OCH_3_); ^13^C NMR (acetone-*d*_6_) δ 234.1 (Cr-CO), 170.7, 170.3, 170.0, 169.7 (O=CO), 109.3 (C1-aryl), 100.9 (C1), 95.3, 95.2, 94.0, 93.8, 93.8 (C-aryl), 72.8 (C3), 72.6 (C5), 71.9 (C2), 70.0 (OCH_2_), 69.3 (C4), 62.7 (C6), 20.6 (3C, OCH_3_), 20.5 (OCH_3_); Anal. calcd for C_24_H_26_CrO_13_ (574.5): C, 50.18; H, 4.56; found: C, 50.10; H, 4.40.

## Supporting Information

File 1 Experimental data.
