# Supplementary material for: Synthesis and structure of tricarbonyl(η6-arene)chromium complexes of phenyl and benzyl D-glycopyranosides
Source: Beilstein J Org Chem. 2012 Jul 11;8:1059–70. doi: 10.3762/bjoc.8.118 (PMC3458723; doi:10.3762/bjoc.8.118)
Supplement: File 1 — Experimental data. [file Beilstein_J_Org_Chem-08-1059-s001.pdf]

Supporting Information

for

Synthesis and structure of tricarbonyl(η^6-arene)chromium complexes of phenyl and benzyl D-glycopyranosides

Thomas Ziegler* and Ulrich Heber

Address: Institute of Organic Chemistry, University of Tuebingen, Auf der Morgenstelle 18, 72076 Tuebingen, Germany

Email: Thomas Ziegler* - thomas.ziegler@uni-tuebingen.de

*Corresponding author

Experimental data

General

All solvents were dried and distilled prior to their use. Reactions were performed under Ar and monitored by TLC on Polygram Sil G/UV silica gel plates from Machery & Nagel. Detection was effected by charring with H_2SO_4 (5% in EtOH) or by inspection of the TLC plates under UV light. Reactions involving $Cr(CO)_6$ or chromium complexes were performed in brown glassware or in the dark. NMR spectra were recorded on a Bruker ARX 250 spectrometer at 250 MHz for proton spectra and 62.5 MHz for carbon spectra, on a Bruker Avance 400 spectrometer at 400 MHz for proton spectra and 100 MHz for carbon spectra and on a Bruker AMX 600 spectrometer at 600 MHz for proton spectra and 150 MHz for carbon spectra. Tetramethylsilane was used at the internal standard. Chemical shifts δ are given in ppm and coupling constants in Hz. All NMR spectra were treated as first-order spectra. HRMS was performed on a Bruker Daltonics APEX 2 FT–ICR spectrometer. FAB MS was

performed on a Finnigan MAT TSQ 70 spectrometer and ionization with Xe. IR spectra were recorded with a Bruker Tensor 27 IR spectrometer. UV spectra were recorded with a Shimadzu UV 2102 PC spectrometer. Elemental analyses were performed on a Hekatech Euro 3000 CHN analyzer. Optical rotations were measured with a Perkin-Elmer Polarimeter 341. Melting points were determined with a Büchi B-540 apparatus and are uncorrected. Preparative chromatography was performed on silica gel (0.032–0.063 mm) from Machery & Nagel using different mixtures of solvents as eluent.

Single-crystal X-ray diffraction was performed on a STOE IPDS one-circle diffractometer (Mo Kα radiation at λ = 71.073 pm) at 220 K. Structure solution and structure refinement was carried out using the programs SHELXS and SHELXL [1]. Structures were refined with the program PLATON [2] and visualized with the program ORTEP-3 [3,4].

Starting materials

The following glycosides **1** were prepared according to literature procedures. Benzyl 2,3,4,6-tetra-*O*-acetyl-β-D-glucopyranoside (**1a**) [5], benzyl 2,3,4,6-tetra-*O*-acetyl-α-D-glucopyranoside (**1b**) [6], phenyl 2,3,4,6-tetra-*O*-acetyl-β-D-glucopyranoside (**1c**) [7], phenyl 2,3,4,6-tetra-*O*-methyl-β-D-glucopyranoside (**1d**) [8], phenyl 2,3,4,6-tetra-*O*-acetyl-α-D-glucopyranoside (**1e**) [9], 3,4,6-tri-O-acetyl-1,2-(1-phenoxy-1-ethylidene)-β-D-mannopyranose (**1f**) [7], phenyl 2,3,4,6-tetra-*O*-acetyl-β-D-mannopyranoside (**1g**) [10], 2,3,4,6-tetra-*O*-acetyl-1-*O*-benzoyl-β-D-glucopyranoside (**1h**) [11], *N*-phenyl-2,3,4,6-tetra-*O*-acetyl-D-glucopyranosylamine (**1i**) [12], phenyl 2,3,4,6-tetra-*O*-acetyl-1-thio-β-D-glucopyranoside (**1j**) [13], 2,3,4,6-tetra-*O*-acetyl-β-D-glucopyranosylbenzene (**1k**) [14], 2-methylphenyl 2,3,4,6-tetra-*O*-acetyl-β-D-glucopyranoside (**1m**) [15], 2-(2,3,4,6-tetra-*O*-acetyl-β-D-glucopyranosyl)methylbenzene (**1q**) [16].

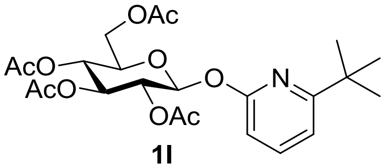

1l

6-*tert*-Butyl-2-pyridyl 2,3,4,5-tetra-*O*-acetyl-β-D-glucopyranoside (1l)

A suspension of acetobromoglucose (2.7 g, 6.6 mmol), 6-*tert*-butyl-2-hydroxypyridine [17] (1.0 g, 6.6 mmol), $Hg(CN)_2$ (1.67 g, 6.6 mmol), dry $CaSO_4$ (4 g) and a catalytic amount of $HgBr_2$ (ca. 100 mg) in MeCN (50 mL) was stirred at rt for 72 h. After the addition of chloroform (100 mL) the mixture was filtered and the filtrate successively washed with aqueous NaI solution (1 M, 3 × 100 mL) and saturated aqueous NaCl solution (1 × 100 mL), dried with Na_2SO_4, filtered and concentrated. Chromatography of the residue with *n*-hexane/ethyl acetate 2:1 and recrystallization from ethanol gave **1l** (1.3 g, 41%) as colorless crystals: Mp 51–53 °C; $[\alpha]_D$ = +5.3 (*c* 1.0, chloroform); ^{1}H NMR ($CDCl_3$) δ 7.54 (t, 1H, H-aryl), 6.98 (d, 1H, H-aryl), 6.59 (d, 1H, H-aryl), 6.21 (d, 1H, $J_{1,2}$ = 8.0 Hz, 1-H), 5.38 (t, 1H, $J_{3,4}$ = 9.1 Hz, 3-H), 5.33 (t, 1H, $J_{2,3}$ = 9.4 Hz, 2-H); 5.18 (t, 1H, $J_{4,5}$ = 9.9 Hz, 4-H), 4.25 (dd, 1H, 6a-H), 4.11 (dd, 1H, 6b-H), 3.94–3.90 (m, 1H, 5-H), 2.05, 2.04, 2.02, 2.00 (4s, 12H, $COCH_3$), 1.32 (s, 9H, $C(CH_3)_3$); ^{13}C NMR ($CDCl_3$) δ 170.7, 170.3, 169.4, 169,4 (4C, O$\underline{C}$OCH_3), 167.1, 159.9, 139.5, 113.5, 108.1 (5C, C-aryl), 93.2 (C1), 73.3 (C3), 72.3 (C5), 70.5 (C4), 68.4 (C2), 62.0 (C6), 37.2 ($\underline{C}(CH_3)_3$), 29.9 (3C, $C(\underline{C}H_3)_3$), 20.6 (4C, $CO\underline{C}H_3$); FT–ICR MS Calcd for $C_{23}H_{32}NO_{10}$ $[M + H]^+$ *m*/*z*: 482.20207; found *m*/*z*: 482.20097.

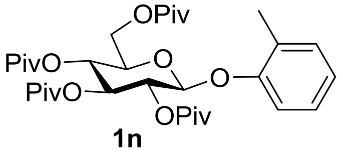

1n

2-Methylphenyl 2,3,4,6-tetra-*O*-pivaloyl-β-D-glucopyranoside (1n)

A solution of **1m** [15] (684 mg, 1.56 mmol) in MeOH (20 mL) containing a catalytic amount of NaOMe (one drop of a 1 M solution in MeOH) was stirred at rt for 2 h and concentrated.

The residue was dissolved in pyridine (20 mL) and cooled to 0 °C. Pivaloyl chloride (1.6 mL, 13 mml) was slowly added with stirring at 0 °C, the solution warmed to rt and stirred for another 12 h. Ethanol and toluene were evaporated several times from the solution until no pyridine and pivaloyl chloride could be detected anymore. The residue was crystallized from ethanol to give **1n** (810 mg, 86%) as colorless needles: Mp 135 °C; $[\alpha]_D$ = −18.1 (*c* 1.0, $CHCl_3$); ^{1}H NMR ($CDCl_3$) δ 7.14–7.07 (m, 2H, H-aryl), 6.96–6.91 (m, 2H, H-aryl), 5.44 (t, 1H, $J_{3,4}$ = 9.4 Hz, 3-H), 5.39 (t, 1H, $J_{2,3}$ = 9.6 Hz, 2-H), 5.18 (t, 1H, 4-H), 5.14 (d, 1H, $J_{1,2}$ = 7.8 Hz, 1-H), 4.25 (d, 1H, 6a-H), 4.05 (dd, 1H, 6b-H), 3.93–3.89 (m, 1H, 5-H), 2.17 (s, 3H, Ph-CH_3), 1.21, 1.18, 1.14, 1.14 (4 s, 36H, $C(CH_3)_3$); ^{13}C NMR ($CDCl_3$) δ 178.0, 177.1, 176.5, 176.4, (4C, O=CO), 154.8, 131.0, 127.7, 126.6, 113.8 (6C, C-aryl), 98.8 (C1), 72.4 (C5), 72.2 (C3), 71.0 (C2), 68.2 (C6), 38.8, 38.7 (4C, $CO\underline{C}(CH_3)_3$), 27.1, 27.0 (12C, $COC(\underline{C}H_3)_3$), 16.0 (Ph-CH_3); Anal. Calcd for $C_{33}H_{50}O_{10}$ (606.7): C, 65.32; H, 8.31; found: C, 65.23; H, 8.33.

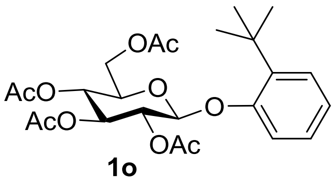

1o

2-*tert*-Butylphenyl 2,3,4,6-tetra-*O*-acetyl-β-D-glucopyranoside (1o)

BF_3-etherate (4.5 mL, 33 mmol) was slowly added at rt to a stirred solution of pentaacetylglucose (13 g, 30 mmol) and 2-*tert*-butylphenol (5 mL, 30 mmol) in dichloromethane (100 mL). The solution was stirred for an additional 24 h, washed with water (3 × 50 mL) and sat. aqueous $NaHCO_3$ solution (3 × 50 mL), dried with Na_2SO_4, filtered and concentrated. Crystallization of the residue from ethanol gave **1o** (4.5 g, 31%) as colorless crystals: Mp 193–195 °C; $[\alpha]_D$ = −33.8 (*c* 1.0, chloroform); ^{1}H NMR ($CDCl_3$) δ 7.31 (dd, 1H, H-aryl), 7.16 (ddd, 1H, H-aryl), 6.99 (t, 2H, H-aryl), 5.39 (t, 1H, $J_{2,3}$ = 9.1 Hz, 2-H), 5.32 (t, 1H, $J_{3,4}$ = 9.0 Hz, 3-H), 5.27 (d, 1H, $J_{1,2}$ = 7.6 Hz, 1-H), 5.19 (t, 1H, 4-H), 4.27 (dd, 1H, 6a-H), 4.18 (dd, 1H, 6b-H), 3.94–3.89 (m, 1H, 5-H), 2.06, 2.05, 2.03, 2.00 (4s, 12H, OCH_3), 1.33

(s, 9H, $C(CH_3)_3$); ^{13}C NMR ($CDCl_3$) δ 170.5, 170.3, 169.4, 169.3 (4C, O=CO), 155.2, 138.6, 127.1, 127.0, 122.5, 114.0 (6C, C-aryl), 97.3 (C1), 73.2 (C3), 71.9 (C5), 71.3 (C2), 68.4 (C4), 62.1 (C6), 34.7 ($\underline{C}(CH_3)_3$), 29.8 (3C, $C(\underline{C}H_3)_3$), 20.7, 20.6 (4C, $CO\underline{C}H_3$); Anal. Calcd for $C_{24}H_{32}O_{10}$ (480.5): C, 59.99; H, 6.71; found: C, 59.32; H, 6.79. FT–ICR MS Calcd for $C_{24}H_{32}NaO_{10}$ $[M + Na]^+$ *m*/*z*: 503.18877; found *m*/*z*: 503.18660.

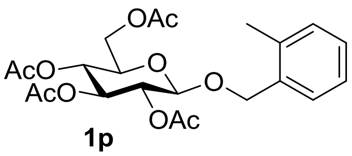

1p

2-Methylbenzyl 2,3,4,5-tetra-*O*-acetyl-β-D-glucopyranoside (1p)

A suspension of acetobromoglucose (5 g, 12.2 mmol), 2-methylbenzylalcohol (1.5 g. 12. mmol), $Hg(CN)_2$ (3 g, 12.2 mmol), dry $CaSO_4$ (4 g) and a catalytic amount of $HgBr_2$ (ca. 100 mg) in MeCN (50 mL) was stirred at rt for 72 h and worked up as described for the preparation of **1l**. Chromatography of the residue with *n*-hexane/ethyl acetate 2:1 and crystallization from ethanol gave **1p** (1.4 g, 25%) as colorless crystals: Mp 55 °C; $[\alpha]_D$ = −50.4 (*c* 1.0, chloroform); ^{1}H NMR ($CDCl_3$) δ 7.25–7.15 (m, 4H, H-aryl), 5.16 (t, 1H, $J_{3,4}$ = 9.1 Hz, 3-H), 5.10 (t, 1H, $J_{4,5}$ = 9.6 Hz, 4-H), 5.05 (t, 1H, $J_{2,3}$ = 9.6 Hz, 2-H), 4.90 (d, 1H, CH_2), 4.63 (d, 1H, CH_2) 4.49 (d, 1H, $J_{1,2}$ = 7.8 Hz, 1-H), 4.27 (dd, 1H, 6a-H), 4.18 (d, 1H, 6b-H), 3.68–3.63 (m, 1H, 5-H), 2.30 (s, 3H, Ph-CH_3), 2.16, 2.11, 1.99, 1.97 (4 s, 12H, $COCH_3$); ^{13}C NMR ($CDCl_3$) δ 170.6, 170.2, 169.4, 169.2 (4 C, O=CO), 137.0, 134.2, 130.3, 129.0, 128.4, 125.8 (6 C, C-aryl), 98.7 (C1), 72.8 (C3), 71.7 (C5), 71.2 (C2), 69.1 (Ph-CH_2), 68.4 (C4), 61.9 (C6), 30.9 (Ph-CH_3), 28.6, 20.7, 20.5 (4C, $CO\underline{C}H_3$); Anal. Calcd for $C_{22}H_{28}O_{10}$ (452.5): C, 58.40; H, 6.24; found: C, 58.42; H, 6.21.

Chromium complexes

General procedure: A solution of glycoside **1** (1 mol equiv) and $Cr(CO)_6$ (1 mol equiv) in di-*n*-butylether/THF 9:1 was heated in the dark under Ar at 140 °C until TLC indicated complete consumption of **1** and then concentrated. Chromatography of the residue under Ar with *n*-hexane/ethyl acetate 2:1 and immediate concentration of the fractions containing the chromium complex gave **2**. Crystalline complexes **2** were slowly recrystallized from ethanol. Suitable crystals were submitted to X-ray crystallography.

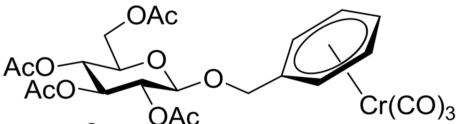

2a

Tricarbonyl[(2,3,4,6-tetra-*O*-acetyl-β-D-glucopyranosyloxymethyl)-η^6-benzene]chromium (2a)

Treatment of **1a** (3.0 g, 6.8 mmol) and $Cr(CO)_6$ (1.50 g, 6.8 mmol) in di-*n*-butylether/THF (100 mL) for 96 h according to the general procedure afforded **2a** (1.14 g, 29%) as yellow triclinic crystals: Mp 140–141 °C (EtOH); $[\alpha]_D = -11.0$ (*c* 1.0, toluene); IR (KBr): 1952 cm^{-1}; 1895 cm^{-1}; FAB MS: *m*/*z* 597 $[M + Na]^+$, *m*/*z* 574 $[M]^+$, *m*/*z* 490 $[M - 3CO]^+$; ^1H NMR (acetone-d_6) δ 5.74–5.56 (m, 5H, H-aryl), 5.28 (t, 1H, 3-H), 5.06 (t, 1H, 4-H), 4.98 (t, 1H, 2-H), 4.98 (dd, 1H, $J_{1,2}$ = 7.3 Hz, 1-H), 4.68–4.45 (dd, 2H, OCH_2Ph), 4.27 (dd, 1H, 6a-H), 4.16 (dd, 1H, 6b-H), 4.01 (m, 1H, 5-H), 2.06–1.94 (m, 12H, OCH_3); ^{13}C NMR (acetone-d_6) δ 234.1 (Cr-CO), 170.7, 170.3, 170.0, 169.7 (O=CO), 109.3 (C1-aryl), 100.9 (C1), 95.3, 95.2, 94.0, 93.8, 93.8 (C-aryl), 72.8 (C3), 72.6 (C5), 71.9 (C2), 70.0 (OCH_2), 69.3 (C4), 62.7 (C6), 20.6 (3C, OCH_3), 20.5 (OCH_3); Anal. Calcd for $C_{24}H_{26}CrO_{13}$ (574.5): C, 50.18; H, 4.56; Found: C, 50.10; H, 4.40.

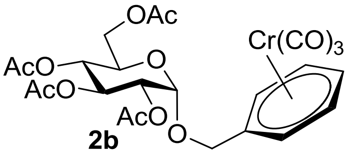

Tricarbonyl[(2,3,4,6-tetra-*O*-acetyl-α-D-glucopyranosyloxymethyl)-η[6]-benzene]chromium (2b)

Treatment of **1b** (3.0 g, 6.8 mmol) and $Cr(CO)_6$ (1.50 g, 6.8 mmol) in di-*n*-butylether/THF (100 mL) for 90 h according to the general procedure afforded **2b** (3.4 g, 87%) as yellow monoclinic crystals: Mp 119–120 °C (EtOH); $[\alpha]_D$ = +107.0 (*c* 1.0, toluene); IR (KBr): 1960 cm^{-1}; 1888 cm^{-1}; FAB MS: *m*/*z* 597 $[M + Na]^+$, *m*/*z* 574 $[M]^+$, *m*/*z* 490 $[M - 3CO]^+$; ^1H NMR (acetone-d_6) δ 5.74–5.61 (m, 5H, H-aryl), 5.50 (t, 1H, $J_{2,3} = J_{3,4}$ = 9.9 Hz, 3-H), 5.27 (d, 1H, $J_{1,2}$ = 3.8 Hz, 1-H), 5.08 (t, 1H, $J_{3,4} = J_{4,5}$ = 9.9 Hz, 4-H), 4.91 (dd, 1H, 2-H), 4.61; 4.41 (dd, 2H, OCH_2Ph), 4.23 (dd, 1H, 6a-H), 4.15 (m, 1H, 5-H), 4.10 (dd, 1H, 6b-H), 2.03–1.96 (m, 12H, OCH_3); ^{13}C NMR (acetone-d_6) δ 234.1 (Cr-CO), 170.7, 170.4, 170.2, 170.7 (O=CO), 108.8 (C1-aryl), 96.8 (C1), 95.2, 95.2, 94.3, 94.2, 94.0 (C-aryl), 71.3 (C2), 70.6 (OCH_2Ph), 69.3 (C3), 69.3 (C4), 68.7 (C5), 62.6 (C6), 20.6, 20.6 ($COCH_3$); Anal. Calcd for $C_{24}H_{26}CrO_{13}$ (574.5): C, 50.18; H, 4.56; found: C, 50.42; H, 4.76.

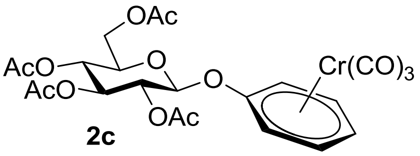

Tricarbonyl[(2,3,4,6-tetra-*O*-acetyl-β-D-glucopyranosyloxy)-η[6]-benzene]chromium (2c)

Treatment of **1c** (3.0 g, 7.1 mmol) and $Cr(CO)_6$ (1.60 g, 7.3 mmol) in di-*n*-butylether/THF (100 mL) for 80 h according to the general procedure afforded **2c** (1.14 g, 29%) as yellow triclinic crystals: Mp 162–163 °C (EtOH); $[\alpha]_D$ = −47.2 (*c* 1.0, toluene); IR (KBr): 1955 cm^{-1}, 1884 cm^{-1}; FAB MS: *m*/*z* 583 $[M + Na]^+$, *m*/*z* 560 $[M]^+$, *m*/*z* 476 $[M - 3CO]^+$; ^1H NMR (acetone-d_6) δ 5.86–5.45 (m, 5H, H-aryl), 5.41 (d, 1H, $J_{1,2}$ = 8.6 Hz, 1-H), 5.23–5.12 (m, 3H, 2-H, 3-H, 4-H), 4.33–4.19 (m, 3H, 5-H, 6a-H, 6b-H), 2.04–1.97 (m, 12H, OCH_3); ^{13}C NMR

(acetone-d_6) δ 234.2 (Cr-CO), 170.8, 170.3, 170.0; 169.8 (O=CO), 140.5 (C1-aryl), 98.0 (C1), 97.0, 96.1, 88.6, 84.0, 81.1 (C-aryl), 73.0 (C5), 72.7 (C3), 71.3 (C2), 68.9 (C4), 62.5 (C6), 20.6, 20.5 ($COCH_3$); Anal. Calcd for $C_{23}H_{24}CrO_{13}$ (560.4): C, 49.29; H, 4.32; found: C, 49.45; H, 4.27.

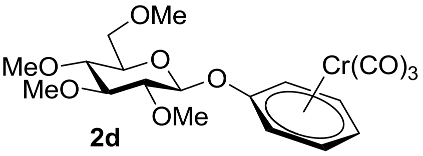

2d

Tricarbonyl[(2,3,4,6-tetra-*O*-methyl-β-D-glucopyranosyloxy)-η^6-benzene]chromium (2d) Treatment of **1d** (1.5 g, 4.8 mmol) and $Cr(CO)_6$ (1.06 g, 4.8 mmol) in di-*n*-butylether/THF (100 mL) for 16 h according to the general procedure afforded **2d** (400 mg, 19%) as yellow monoclinic crystals: Mp 126–129 °C (EtOH); $[\alpha]_D$ = −60.0 (*c* 1.0, toluene); IR (KBr): 1963 cm^{-1}, 1893 cm^{-1}; FAB MS: *m*/*z* 472 $[M + H + Na]^+$, *m*/*z* 448 $[M]^+$, *m*/*z* 364 $[M - 3CO]^+$; ^{1}H NMR (acetone-d_6) δ 5.84–5.77 (m 2H, H-aryl), 5.57 (t, 1H, H-aryl), 5.18 (t, 1H, H-aryl), 4.91 (d, 1H, $J_{1,2}$ = 7.6 Hz, 1-H), 3.64–3.55 (m, 3H, 5-H, 6a-H, 6b-H), 3.24 (t, 1H, $J_{3,4}$ = 8 Hz, 3-H), 3.13 (t, 1H, $J_{4,5}$ = 9.4 Hz, 4-H), 3.08 (t, 1H, $J_{2,3}$ = 8.9 Hz, 2-H), 3.58, 3.56, 3.49, 3.33 (4 s, 12H, $COCH_3$); ^{13}C NMR (acetone-d_6) δ 234.3 (3 C, Cr-CO), 141.2 (C1-aryl), 101.1 (C1), 96.7, 96.1, 88.4, 83.3, 81.1 (C-aryl), 86.8 (C3), 84.1 (C2), 79.7 (C4), 75.5 (C5). 71.8 (C6), 60.9, 60.6, 60.4, 59.2 (4 C, $COCH_3$); Anal. Calcd for $C_{19}H_{24}CrO_9$ (448.4): C, 50.89; H, 5.40; found: C, 51.39; H, 5.42.

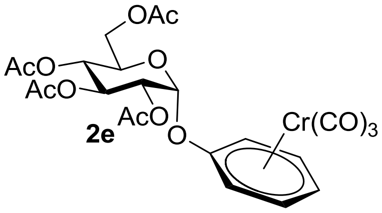

2e

Tricarbonyl[(2,3,4,6-tetra-*O*-acetyl-α-D-glucopyranosyloxy)-η^6-benzene]chromium (2e)

Treatment of **1e** (1.5 g, 3.5 mmol) and $Cr(CO)_6$ (0.78 g, 3.5 mmol) in di-*n*-butylether/THF (100 mL) for 70 h according to the general procedure afforded **2e** (1.03 g, 53%) as yellow orthorhombic crystals: Mp 134–137 °C (EtOH); $[\alpha]_D$ = +134.1 (*c* 1.0, toluene); IR (KBr): 1968 cm^{-1}, 1896 cm^{-1}; FAB MS: *m*/*z* 583 [M + Na]$^+$, *m*/*z* 504 [M − 2CO]$^+$, *m*/*z* 476 [M − 3CO]$^+$; ^{1}H NMR (acetone-d_6) δ 5.91–5.71 (m, 4H, H-aryl), 5.28 (t, 1H, H-aryl), 5.67 (d, 1H, $J_{1,2}$ = 3.4 Hz, 1-H), 5.53 (t, 1H, $J_{3,4}$ = 9.7 Hz, 3-H), 5.15 (t, 1H, $J_{4,5}$ = 9.8 Hz, 4-H), 5.10 (dd, 1H, $J_{2,3}$ = 10.6 Hz, 2-H), 4.26 (dd, 1H, 6a-H), 4.18–4.10 (m, 2H, 5-H, 6b-H), 2.02, 2.01, 2.00, 1.99 (4 s, 12H, $COCH_3$); ^{13}C NMR (acetone-d_6) δ 234.1 (3 C, Cr-CO), 170.3, 170.1, 170.0, 169.7 (4 C, O=CO), 140.6 (C1-aryl), 96.3 (C1), 97.3, 96.7, 89.5, 84.9, 82.3 (C-aryl), 70.6 (C3), 70.5 (C2), 70.4 (C5), 69.3 (C4), 62.8 (C6), 20.6, 20.5 (4 C, $COCH_3$); Anal. Calcd for $C_{23}H_{24}CrO_{13}$ (560.4): C, 49.29; H, 4.32; found: C, 49.23; H, 4.25.

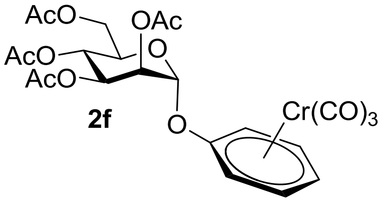

2f

Tricarbonyl[(2,3,4,6-tetra-*O*-acetyl-α-D-mannopyranosyloxy)-η^6-benzene]chromium (2f)

Treatment of **1f** (1.0 g, 2.4 mmol) and $Cr(CO)_6$ (0.52 g, 2.4 mmol) in di-*n*-butylether/THF (100 mL) for 42 h according to the general procedure afforded **2f** (0.63 g, 47%) as a yellow crystals which were not suitable for X-ray crystallography: Mp 129–131 °C (EtOH); $[\alpha]_D$ = +62.8 (c 1.0, toluene); IR (KBr): 1957 cm^{-1}, 1867 cm^{-1}; ^{1}H NMR (acetone-d_6) δ 5.89 (d, 2H, H-aryl), 5.75 (d, 1H, H-aryl), 5.70 (d, 1H, H-aryl), 5.59 (s, 1H, 1-H), 5.40–5.25 (m, 4H, 2-H,

3-H, 4-H, H-aryl), 4.29–4.16 (m, 3H, 5-H, 6a-H, 6b-H), 2.14, 2.04, 2.03, 1.97 (4 s, 12H, $COCH_3$); ^{13}C NMR (acetone-d_6) δ 234.4 (3 C, Cr-CO), 170.6, 170.5, 170.1, 170.0 (4C, O=CO), 140.3 (C1-aryl), 97.6 (C1), 96.7, 96.3, 89.1, 83.8, 82.3 (C-aryl), 71.0 (C5), 69.4, 69.2, 66.0 (C2, C3, C4), 62.6 (C6), 20.6, 20.5 (4 C, $COCH_3$); FT–ICR MS: Calcd for $C_{23}H_{24}CrNaO_{13}$ $[M + Na]^+$ *m*/*z*: 583.05031; found *m*/*z*: 583.04976

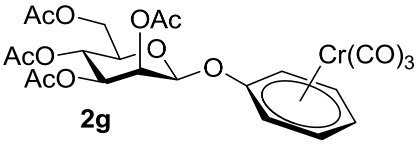

Tricarbonyl[(2,3,4,6-tetra-*O*-acetyl-β-D-mannopyranosyloxy)-η^6-benzene]chromium (2g)

Treatment of **1g** (1.0 g, 2.4 mmol) and $Cr(CO)_6$ (0.52 g, 2.4 mmol) in di-*n*-butylether/THF (100 mL) for 42 h according to the general procedure (without crystallization from EtOH) afforded **2g** (0.47 g, 35%) as a yellow amorphous solid: $[\alpha]_D$ = −84.7 (*c* 1.0, toluene); ^{1}H NMR (acetone-d_6): broad multiplets between 6 and 4 ppm. ^{13}C NMR (acetone-d_6) δ 234.3 (3C, Cr-CO), 170.7, 170.4, 170.2, 170.1 (4C, O=CO), 140.8 (C1-aryl), 97.3, 96.5, 96.2, 88.2, 83.6, 80.9 (C1, C-aryl), 73.0 (C5), 71.1, 69.2, 66.5 (3 C, C2, C3, C4), 63.0 (C6), 20.6, 20.5, 20.4 (4 C, $COCH_3$); FT–ICR MS Calcd for $C_{23}H_{24}CrNaO_{13}$ $[M + Na]^+$ *m*/*z*: 583.05031; found *m*/*z*: 583.05025

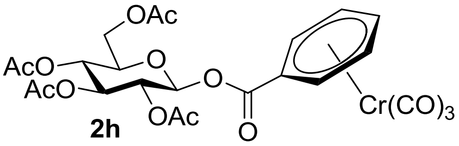

Tricarbonyl[(2,3,4,6-tetra-*O*-acetyl-β-D-glucopyranosyloxycarbonyl)-η^6-benzene]chromium (2h)

Treatment of **1h** (1.0 g, 2.2 mmol) and $Cr(CO)_6$ (487 mg, 2.2 mmol) in di-*n*-butylether/THF (100 mL) for 67 h according to the general procedure afforded **2h** (390 mg, 30%) as orange

crystals, which were not suitable for X-ray crystallography: Mp 117 °C (EtOH); $[\alpha]_D$ = +6.5 (*c* 1.0, toluene); IR (KBr): 1975 cm^{-1}, 1902 cm^{-1}; FAB–MS: *m*/*z* 611 $[M + Na]^+$, *m*/*z* 506 $[M - 3CO]^+$; ^1H NMR (acetone-d_6) δ 6.28 (d, 2H, H-aryl), 6.21 (d, 1H, H-aryl), 6.08 (d, 1H, $J_{1,2}$ = 8.3 Hz, 1-H), 5.70 (t, 2H, H-aryl), 5.48 (t, 1H, $J_{3,4}$ = 9.6 Hz, 3-H), 5.19 (dd, 1H, $J_{2,3}$ = 8.5 Hz, 2-H), 5.14 (dd, 1H, $J_{4,5}$ = 9.8 Hz, 4-H), 4.23 (m, 1H, 5-H), 4.31 (dd, 1H, 6a-H), 4.11 (dd, 1H, 6b-H), 2.02, 2.01, 2.00, 1.98 (4 s, 12H, $COCH_3$); ^{13}C NMR (acetone-d_6) δ 232.1 (3C, Cr-CO), 170.6, 170.2, 170.0, 169.9 (4C, O=COMe), 164.6 (1C, O=COPh), 97.7, 96.7, 96.3, 92.3, 92.0 (C-aryl), 93.4 (C1), 89.3 (C1), 73.3 (C5), 73.0 (C3), 70.9 (C2), 68.9 (C4), 62.4 (C6), 20.6, 20.5 (4C, $CO\underline{C}H_3$); Anal. Calcd for $C_{24}H_{24}CrO_{14}$ (588.4): C, 48.99; H, 4.11; found: C, 48.94; H, 4.09.

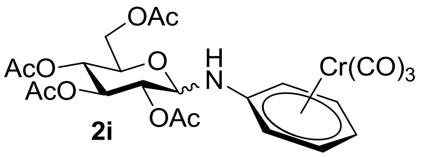

Tricarbonyl[(2,3,4,6-tetra-*O*-acetyl-D-glucopyranosylamino)-η^6-benzene]chromium (2i)

Treatment of **1i** (1.0 g, 2.4 mmol) and $Cr(CO)_6$ (0.52 g, 2.4 mmol) in di-*n*-butylether/THF (100 mL) for 24 h according to the general procedure (without crystallization from EtOH) afforded a 1:2 anomeric α/β-mixture of **2i** (0.60 g, 46%) as a yellow amorphous solid: IR (KBr): 1954 cm^{-1}, 1867 cm^{-1}; FAB–MS: *m*/*z* 559 $[M]^+$, *m*/*z* 475 $[M - 3CO]^+$; ^1H NMR (acetone-d_6): broad multiplets between 6 and 4 ppm. ^{13}C NMR (acetone-d_6) δ 235.2, 235.1 (Cr-CO), 170.7, 170.6, 170.3, 170.2, 170.0, 169.9 (O=CO), 131.9, 131.4, 98.1, 97.7, 86.1, 85.4, 82.2, 80.3, 79.8, 79.8, 78.4, 77.5, 73.8, 72.8, 71.5, 70.6, 69.6, 69.5, 69.2, 67.8, 66.0, 62.8, 62.6 (24 C, C-aryl, C1-6), 20.6, 20.5 ($CO\underline{C}H_3$); Anal. Calcd for $C_{23}H_{25}CrNO_{12}$ (559.4): C, 48.38; H, 4.50; N, 2.50; found: C, 49.36; H, 4.57; N, 2.41.

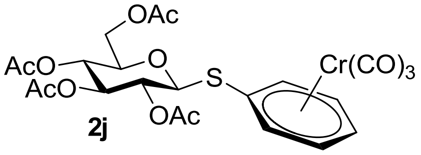
2j

Tricarbonyl[(2,3,4,6-tetra-*O*-acetyl-β-D-glucopyranosylthio)-η[6]-benzene]chromium (2j)

Treatment of **1j** (1.0 g, 2.3 mmol) and $Cr(CO)_6$ (0.50 g, 2.3 mmol) in di-*n*-butylether/THF (100 mL) for 24 h according to the general procedure afforded **2j** (0.64 g, 49%) as yellow triclinic crystals: Mp 123 °C (EtOH); $[\alpha]_D$ = +143.6 (*c* 1.0, toluene); IR (KBr): 1972 cm^{-1}, 1888 cm^{-1}; FAB–MS: *m*/*z* 599 $[M + Na]^+$, *m*/*z* 576 $[M]^+$, *m*/*z* 492 $[M - 3CO]^+$; ^1H NMR (acetone-d_6) δ 5.90−5.66 (m, 5H, H-aryl), 5.35 (t, 1H, $J_{3,4}$ = 9.4 Hz, 3-H), 5.06 (d, 1H, $J_{1,2}$ = 10.1 Hz, 1-H), 5.02 (t, 1H, 4-H), 4.96 (t, 1H, $J_{2,3}$ = 10.1 Hz, 2-H), 4.20 (d, 2H, 6a-H, 6b-H), 4.10–4.05 (m, 1H, 5-H), 2.06, 2.05, 2.00, 1.95 (4 s, 12H, $COCH_3$); ^{13}C NMR (acetone-d_6) δ 233.4 (3C, Cr-CO), 170.7, 170.2, 170.0, 169.9 (4C, O=CO) 102.8 (C1-aryl), 100.7, 100.1, 94.4, 94.2, 94.0 (C-aryl), 85.5 (C1), 76.4 (C5), 74.2 (C3), 70.4 (C2), 68.9 (C4), 62.8 (C6), 20.7, 20.6, 20.5, 20.4 (4C, $COCH_3$); Anal. Calcd for $C_{23}H_{24}CrO_{12}S$ (576.5): C, 47.92; H, 4.20; S, 5.56; found: C, 47.85; H, 4.10; S, 5.54.

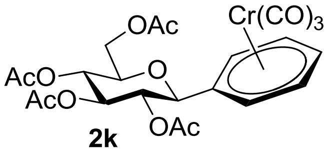
2k

Tricarbonyl[(2,3,4,6-tetra-*O*-acetyl-β-D-glucopyranosyl)-η[6]-benzene]chromium (2k)

Treatment of **1k** (3.0 g, 7.4 mmol) and $Cr(CO)_6$ (1.62 g, 7.4 mmol) in di-*n*-butylether/THF (100 mL) for 80 h according to the general procedure afforded **2k** (3.24 g, 81%) as yellow orthorhombic crystals: Mp 100 °C decomp. (EtOH); $[\alpha]_D$ = −48.9 (*c* 1.0, toluene); IR (KBr): 1969 cm^{-1}, 1889 cm^{-1}; FAB–MS: *m*/*z* 567 $[M + Na]^+$, *m*/*z* 545 $[M + H]^+$, *m*/*z* 460 $[M - 3CO]^+$; ^1H NMR (acetone-d_6) δ 5.73–5.55 (m, 5H, H-aryl), 5.38 (t, 1H, 3-H), 5.14 (t, 1H, 4-H), 5.0 (t, 1H, 2-H), 4.34 (dd, 1H, 6°-H) 4.30 (d, 1H, $J_{1,2}$ = 9.9 Hz, 1-H) 4.11–4.07 (m, 2H, 5-

H, 6b-H), 2.02–1.94 (m, 12H, OCH_3); ^{13}C NMR (acetone-d_6) δ 234.0 (Cr-CO), 170.8, 170.3, 170.1, 169.7 (O=CO), 109.8 (C1-aryl), 94.3, 94.2, 93.9, 93.8, 91.6 (C-aryl), 77.6 (C1), 76.6 (C5), 74.3 (C3), 73.8 (C2), 69.5 (C4), 63.1 (C6), 20.6, 20.5 (CO<u>C</u>H3); Anal. Calcd for $C_{23}H_{24}CrO_{12}$ (544.0): C, 50.74; H, 4.44; found: C, 50.64; H, 4.47.

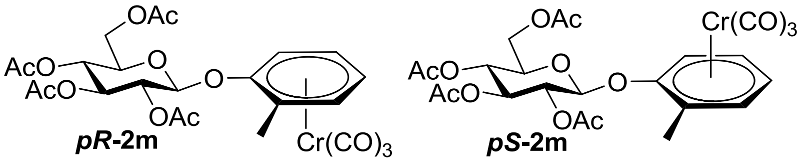

(*pR*)-Tricarbonyl[1-methyl-2-(2,3,4,6-tetra-*O*-acetyl-β-D-glucopyranosyloxy)-η^6-benzene]chromium (*pR*-2m) and (*pS*)-tricarbonyl[1-methyl-2-(2,3,4,6-tetra-*O*-acetyl-β-D-glucopyranosyloxy)-η^6-benzene]chromium (*pS*-2m)

Treatment of **1m** (2.5 g, 5.7 mmol) and $Cr(CO)_6$ (1.25 g, 5.7 mmol) in di-*n*-butylether/THF (100 mL) for 70 h according to the general procedure without crystallization from EtOH afforded **2m** (2.47 g, 76%) as a 7:3 mixture of diastereomers ***pR*-2m** and ***pS*-2m** as determined by ^{1}H NMR. Slow crystallization of the mixture from EtOH afforded first pure monoclinic crystals of ***pR*-2m**: Mp 194–197 °C (EtOH); $[\alpha]_D$ = −130.5 (*c* 1.0, acetone); IR (KBr): 1956 cm^{-1}, 1873 cm^{-1}; FAB–MS: *m/z* 597 $[M + Na]^+$, *m/z* 574 $[M]^+$, *m/z* 490 $[M - 3CO]^+$; ^{1}H NMR (acetone-d_6) δ 5.81 (d, 1H, H-aryl), 5.74 (d, 1H, H-aryl), 5.65 (t, 1H, H-aryl), 5.49 (t, 1H, $J_{3,4}$ = 9.6 Hz, 3-H), 5.35 (d, 1H, $J_{1,2}$ = 7.9 Hz, 1-H), 5.30 (t, 1H, H-aryl), 5.19 (t, 1H, $J_{2,3}$ = 9.6 Hz, 2-H), 4.96 (t, 1H, $J_{4,5}$ = 9.7 Hz, 4-H), 4.39–4.38 (m, 1H, 5-H), 4.36–4.22 (m, 2H, 6a-H, 6b-H), 2.85 (s, 3H, $PhCH_3$), 2.08, 2.00, 2.00, (3 s, 12H, $COCH_3$); ^{13}C NMR (acetone-d_6) δ 234.5 (3C, Cr-CO), 170.6, 170.5, 170.0, 169.8 (4C, O=CO), 138.3 (C-aryl), 101.0 (C1), 98.7, 97.6, 94.0, 90.0, 81.7 (C-aryl), 72.7, (2 C, C3, C5), 71.2 (C2), 68.9 (C4), 62.4 (C6), 20.6, 20.5 (4C, CO<u>C</u>H3), 16.0 (Ph-CH_3); Anal. Calcd for $C_{24}H_{26}CrO_{13}$ (574.5): C, 50.18; H, 4.56; found: C, 50.34; H, 4.57.

Further fractionating crystallization of **2m** from the mother liquor gave a crystal fraction containg both diastereomers **2m** followed by a small amount of pure triclinic crystals of ***pS*-2m**, the amount of which was too small for characterization. ^{13}C NMR data could be obtained from the spectra of the mixture of diastereomers and the few crystals obtained were suitable for X-ray crystallography. ^{13}C NMR (acetone-d_6) δ 234.5 (3C, Cr-CO), 170.6, 170.5, 170.0, 169.8 (4C, O=CO), 138.4 (C-aryl), 100.3 (C1), 99.8, 96.8, 93.2, 90.9, 84.0 (C-aryl), 72.9, (2C, C3, C5), 71.5 (C2), 69.1 (C4), 62.8 (C6), 20.6, 20.5 (4C, $CO\underline{C}H_3$), 16.0 (Ph-CH_3).

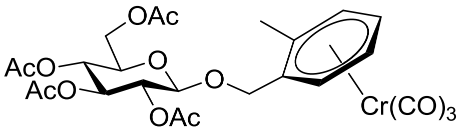

2p

Tricarbonyl[1-methyl-2-(2,3,4,6-tetra-*O*-acetyl-β-D-glucopyranosyloxymethyl)-η^6-benzene]chromium (2p)

Treatment of **1p** (1.4 g, 3.1 mmol) and $Cr(CO)_6$ (0.68 g, 3.1 mmol) in di-*n*-butylether/THF (100 mL) for 15 h according to the general procedure without crystallization from EtOH afforded a 1:1 mixture of diastereomers of **2p** (0.77 g, 42%) as a yellow amorphous solid: IR (KBr): 1950 cm^{-1}, 1870 cm^{-1}; FAB MS: *m*/*z* 588 [M]$^+$, *m*/*z* 504 [M − 3CO]$^+$; ^{1}H NMR (acetone-d_6) δ 5.78 (t, 2H, H-aryl), 5.72–5.66 (m, 2H, H-aryl), 5.57–5.48 (m, 4H, H-aryl), 5.28 (t, 2H, 3-H), 5.09–5.03 (m, 2H, 4-H), 4.97–4.93 (m, 4H, $J_{1,2}$ = 8.1 Hz, 1-H, 2-H), 4.84 (d, 1H, CH_2-H); 4.64 (d, 1H, CH_2), 4.49 (d, 1H, CH_2), 4.40 (d, 1H. CH_2); 4.31–4.25 (m, 2H, 6a-H), 4.18–4.14 (m, 2H, 6b-H), 4.02 (m, 2H, 5-H), 2.25, 2.23 (2 s, 6H, Ph-CH_3), 2.03, 2.00, 1.97, 1.94 (4 s, 24H, $COCH_3$); ^{13}C NMR (acetone-d_6) δ 234.4, 234.3 (6C, Cr-CO), 170.7, 170.7, 170.3, 170.2, 170.0, 169.9, 169.7,169.6 (8C, O=CO), 111.5, 110.5, 106.2, 105.6 (4C, C1-aryl, C2-aryl), 100.8, 100.0 (2C, C1), 97.8, 96.3, 96.1, 96.1, 95.7, 95.6, 92.3, 92.0 (8C, C-aryl), 73.4, 73.3 (2C, C3), 72.5, (2C, C5), 72.0, 71.9 (2C, C2), 69.3, 69.2, 69.0, 67.9 (4C,

CH_2), 62.6 (2 C, C6), 20.6, 20.5 (8C, $CO\underline{C}H_3$), 18.2, 18.0 (Ph-CH_3); Anal. Calcd for $C_{25}H_{28}CrO_{13}$ (588.5): C, 51.02; H, 4.80; found: C, 51.50; H, 4.90.

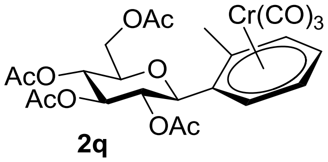

2q

Tricarbonyl[1-methyl-2-(2,3,4,6-tetra-*O*-acetyl-β-D-glucopyranosyl)-η^6-benzene]chromium (2q)

Treatment of **1q** (650 mg, 1.5 mmol) and $Cr(CO)_6$ (338 mg, 1.5 mmol) in di-*n*-butylether/THF (100 mL) for 16 h according to the general procedure without crystallization from EtOH afforded a 1:1 mixture of diastereomers of **2q** (350 mg, 42%) as a yellow amorphous solid: IR (KBr): 1957 cm^{-1}, 1866 cm^{-1}; FAB–MS: *m*/*z* 558 $[M]^+$, *m*/*z* 474 $[M - 3CO]^+$; ^1H NMR (acetone-d_6): broad signals between 6 and 4 ppm. ^{13}C NMR (acetone-d_6) δ 234.3, 234.1 (6C, Cr-CO), 170.7, 170.6, 170.3, 170.1, 169.3 (O=CO), 110.7, 110.7, 108.4, 105.6, 95.9, 95.8, 95.6, 95.5, 94.1, 94.0, 91.5, 91.0, 78.2, 76.6, 76.4, 75.1, 74.8, 74.4, 73.7, 70.1, 69.7, 69.3, 63.3, 63.0 (24C, C-aryl, C1-6), 20.8, 20.6, 20.4, 20.3 ($CO\underline{C}H_3$), 19.4, 18.7 (Ph-CH_3); FT–ICR MS Calcd for $C_{24}H_{26}CrNaO_{12}$ $[M + Na]^+$ *m*/*z*: 581.07216; found *m*/*z*: 581.07208.

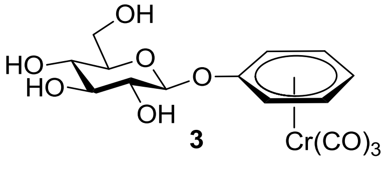

3

Tricarbonyl(β-D-glucopyranosyloxy-η^6-benzene)chromium (3)

A suspension of **2c** (3.2 g, 5.7 mmol) and a catalytic amount of NaOMe (0.1 mL of a 1 M solution in MeOH) in MeOH (100 mL) was stirred at rt under Ar in the dark for 24 h whereupon a clear solution was achieved. The solution was neutralized by the addition of ion

exchange resin (Sephadex, H^+ form), filtered and concentrated to give **3** (2.2 g, 100%) as a yellow amorphous solid: Mp 150 °C decomp. $[\alpha]_D$ = −64.4 (*c* 1.0, methanol); IR (ATR): 1950 cm^{-1}, 1867 cm^{-1}; FAB–MS: *m*/*z* 392 $[M]^+$, *m*/*z* 308 $[M - 3CO]^+$; ^{1}H NMR (methanol-d_4) δ 5.68–5.64 (m, 2H, H-aryl), 5.54–5.52 (d, 2H, H-aryl), 5.07 (t, 1H, H-aryl), 4.78 (d, 1H, $J_{1,2}$ = 7.6 Hz, 1-H), 3.92–3.89 (m, 1H, 6a-H), 3.69 (dd, 1H, 6b-H), 3.45–3.33 (m, 4H, 2-H, 3-H, 4-H, 5-H); ^{13}C NMR (methanol-d_4) δ 234.5 (3C, Cr-CO), 141.7 (C1-aryl), 102.4 (C1), 96.2, 95.8, 88.4, 84.2, 82.4, (C-aryl), 78.2, 77.5, 74.4, 71.1, (4C, C2, C3, C4, C5), 62.4 (C6). FT–ICR MS Calcd for $C_{15}H_{16}CrNaO_9$ $[M + Na]^+$ *m*/*z*: 415.00916; found *m*/*z*: 415.00915.

Enzymatic cleavage of tricarbonyl(β-D-glucopyranosyloxy-η^6-benzene)chromium (3)

β-Glucosidase from almonds (100 mg) and diatomaceous earth (2.5 g) were mixed with water (30 mL), and the mixture was lyophilized and resuspended in citrate/phosphate buffer (0.1 N, pH 5.0, 150 mL) under Ar. To this suspension was added **3** (200 mg, 0.51 mmol), and the mixture was placed on a shaker and shook in the dark at rt for 16 h. Dichloromethane (100 mL) was added and shaking continued for 10 min. The mixture was filtered, the organic layer separated from the filtrate, dried with Na_2SO_4 and concentrated to give tricarbonyl(η^6-phenol)chromium (115 mg, 98%) the NMR spectrum of which was identical to the literature spectrum [18].

X-Ray data

The supplementary crystallographic data for this paper can be obtained free of charge from The Cambridge Crystallographic Data Centre via http://www.ccdc.cam.ac.uk/data_request/cif by using the following numbers: CCDC 870827 for **2a**, CCDC 870828 for **2b**, CCDC 870829 for **2c**, CCDC 870830 for **2d**, CCDC 870831 for **2e**, CCDC 870832 for **2j**, CCDC 870833 for **2k**, CCDC 870834 for ***pR*-2m**, and CCDC 870835 for ***pS*-2m**.

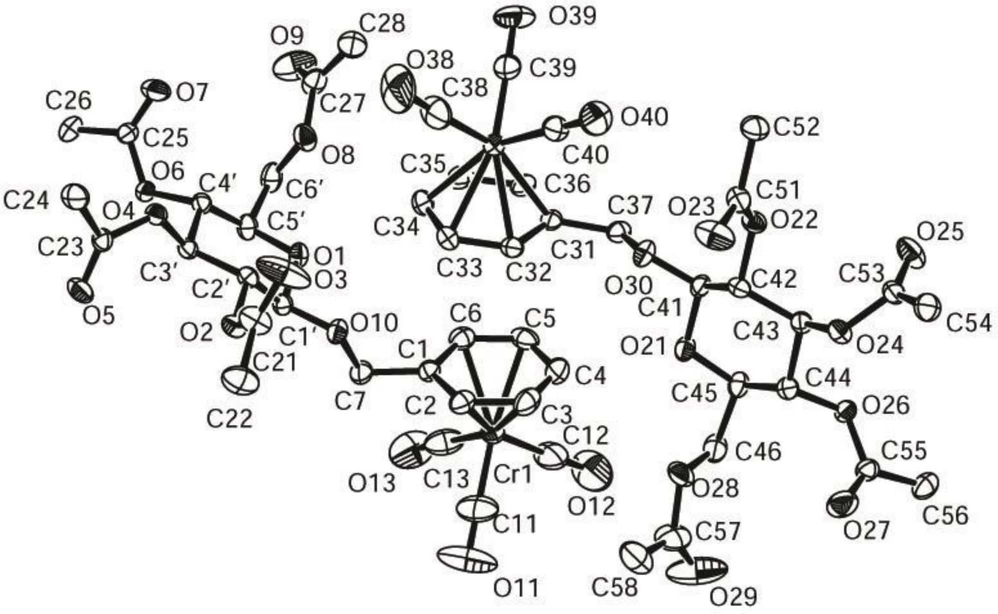

Figure S1: ORTEP-plot for compound **2a** showing 30% probability ellipsoids.

Table S1a: Atomic coordinates for compound **2a**.

	x	y	z	U(eq) ($pm^2 \cdot 10^{-1}$)
Cr(1)	1.2856(1)	1.0594(1)	0.8408(1)	40(1)
O(1)	1.2223(4)	1.4834(3)	1.1247(2)	41(1)
O(2)	1.3180(4)	1.3023(3)	1.0555(2)	42(1)
O(3)	1.5846(3)	1.3000(2)	1.1881(2)	37(1)
O(4)	1.6507(3)	1.5334(2)	1.3074(2)	37(1)
O(5)	1.3180(3)	1.6438(2)	1.3444(2)	36(1)
O(6)	1.2083(6)	1.7306(3)	1.1336(2)	77(1)
O(7)	1.8143(5)	1.3613(3)	1.1284(3)	71(1)
O(8)	1.6651(4)	1.3931(3)	1.3929(2)	63(1)
O(9)	1.4172(5)	1.8359(3)	1.3422(2)	70(1)
O(10)	1.0967(6)	1.9130(4)	1.1703(5)	142(2)
O(11)	1.5250(9)	0.9871(7)	0.7096(4)	139(2)
O(12)	1.2472(6)	0.7909(4)	0.8513(3)	96(2)
O(13)	1.6065(5)	1.0835(4)	0.9689(3)	81(1)
C(1)	1.3096(5)	1.3733(3)	1.1351(2)	37(1)
C(2)	1.4934(5)	1.4107(3)	1.1795(2)	32(1)
C(3)	1.4788(5)	1.4852(3)	1.2665(2)	33(1)
C(4)	1.3620(5)	1.5958(4)	1.2612(2)	34(1)
C(5)	1.1915(6)	1.5592(4)	1.2028(3)	41(1)

C(6)	1.0989(7)	1.6716(5)	1.1819(3)	56(1)
C(7)	1.7414(6)	1.2834(4)	1.1583(3)	40(1)
C(8)	1.8109(7)	1.1615(5)	1.1687(4)	54(1)
C(9)	1.7312(6)	1.4776(4)	1.3687(2)	38(1)
C(10)	1.9084(6)	1.5370(5)	1.3993(4)	50(1)
C(11)	1.3501(5)	1.7662(3)	1.3774(3)	39(1)
C(12)	1.2926(8)	1.8002(5)	1.4621(3)	53(1)
C(13)	1.1951(8)	1.8525(5)	1.1301(5)	76(2)
C(14)	1.320(1)	1.8974(8)	1.0794(5)	93(2)
C(15)	1.1634(6)	1.2283(4)	1.0224(3)	41(1)
C(16)	1.1434(6)	1.1919(4)	0.9287(3)	36(1)
C(17)	1.0280(6)	1.0951(4)	0.8863(3)	45(1)
C(18)	0.9972(7)	1.0648(5)	0.7983(3)	54(1)
C(19)	1.0871(8)	1.1304(5)	0.7512(4)	57(2)
C(20)	1.2091(8)	1.2280(5)	0.7911(3)	55(1)
C(21)	1.2360(7)	1.2593(4)	0.8784(3)	44(1)
C(22)	1.4279(9)	1.0175(6)	0.7606(4)	78(2)
C(23)	1.2628(7)	0.8967(5)	0.8483(3)	58(1)
C(24)	1.4816(7)	1.0735(5)	0.9192(3)	52(1)
Cr(2)	0.7297(1)	1.5605(1)	0.8785(1)	49(1)
O(14)	0.8431(4)	1.1794(3)	0.5836(2)	39(1)
O(15)	0.9733(4)	1.3637(3)	0.6520(2)	41(1)
O(16)	1.1760(3)	1.3715(2)	0.5206(2)	38(1)
O(17)	1.1734(3)	1.1385(2)	0.4015(2)	37(1)
O(18)	0.8209(3)	1.0221(2)	0.3623(2)	36(1)
O(19)	0.8410(5)	0.9311(3)	0.5740(2)	57(1)
O(20)	1.4245(5)	1.3134(4)	0.5864(4)	112(2)
O(21)	1.1516(4)	1.2767(3)	0.3143(2)	52(1)
O(22)	0.9198(5)	0.8308(3)	0.3651(2)	68(1)
O(23)	0.7011(6)	0.7445(3)	0.5427(3)	100(2)
O(24)	0.7656(8)	1.8228(5)	0.8603(6)	157(3)
O(25)	0.538(1)	1.6341(7)	1.0283(5)	196(4)
O(26)	0.3842(7)	1.5416(6)	0.7640(4)	136(2)
C(25)	0.9220(5)	1.2928(3)	0.5724(2)	34(1)
C(26)	1.0807(5)	1.2595(3)	0.5276(2)	33(1)
C(27)	1.0208(5)	1.1832(3)	0.4403(2)	33(1)
C(28)	0.9103(5)	1.0715(4)	0.4456(2)	34(1)
C(29)	0.7717(6)	1.1006(4)	0.5057(3)	43(1)
C(30)	0.6992(7)	0.9841(6)	0.5284(4)	55(1)
C(31)	1.3441(6)	1.3894(4)	0.5545(3)	55(1)

C(32)	1.416(1)	1.5137(6)	0.5471(6)	70(2)
C(33)	1.2250(5)	1.1923(4)	0.3393(2)	36(1)
C(34)	1.3866(8)	1.1352(5)	0.3091(4)	52(1)
C(35)	0.8342(6)	0.9000(3)	0.3285(2)	39(1)
C(36)	0.7360(8)	0.8638(5)	0.2429(3)	47(1)
C(37)	0.8249(8)	0.8097(4)	0.5775(3)	55(1)
C(38)	0.9742(8)	0.7678(5)	0.6291(3)	76(2)
C(39)	0.8320(7)	1.4301(5)	0.6875(3)	50(1)
C(40)	0.8615(6)	1.4406(4)	0.7817(3)	38(1)
C(41)	0.9869(7)	1.5269(5)	0.8319(3)	48(1)
C(42)	1.0123(8)	1.5392(5)	0.9203(4)	61(2)
C(43)	0.9108(9)	1.4662(6)	0.9593(4)	66(2)
C(44)	0.7894(8)	1.3767(5)	0.9107(4)	58(2)
C(45)	0.7641(7)	1.3635(5)	0.8223(3)	49(1)
C(46)	0.749(1)	1.7185(6)	0.8675(6)	93(2)
C(47)	0.606(1)	1.6039(7)	0.9660(5)	116(3)
C(48)	0.5195(9)	1.5472(6)	0.8107(5)	91(2)

Table S1b: Crystal data and structure refinement for **2a**.

Empirical formula	$C_{24}H_{26}CrO_{13}$	
Formula weight	574.45	
Temperature	220(2) K	
Wavelength	71.073 pm	
Crystal system	Triclinic	
Space group	*P*1	
Unit cell dimensions	*a* = 763.9(1) pm	α = 100.99(2)°
	b = 1089.3(2) pm	β = 96.97(2)°
	c = 1614.1(3) pm	γ = 91.03(2)°
Volume	1.3076(4) nm^3	
Z	2	
Density (calculated)	1.459 g/cm^3	
Absorption coefficient	0.504 mm^{-1}	
F(000)	596	
Crystal size	0.40 × 0.26 × 0.06 mm^3	
Theta range for data collection	2.50 to 28.11°	
Index ranges	-10 ≤ h ≤ 10, -14 ≤ k ≤ 14, -21 ≤ l ≤ 21	
Reflections collected	18039	
Independent reflections	11721 [R_{int} = 0.0435]	
Completeness to theta = 28.11°	92.2%	
Absorption correction	None	
Refinement method	Full-matrix least-squares on F^2	

Data/restraints/parameters	11721/3/881
Goodness-of-fit on F^2	0.788
Final R indices [I>2sigma(I)]	R_1 = 0.0414, wR_2 = 0.0826
R indices (all data)	R_1 = 0.0870, wR_2 = 0.0917
Absolute structure parameter	0.00(2)
Largest diff. peak and hole	0.390 and -0.212 eÅ^3

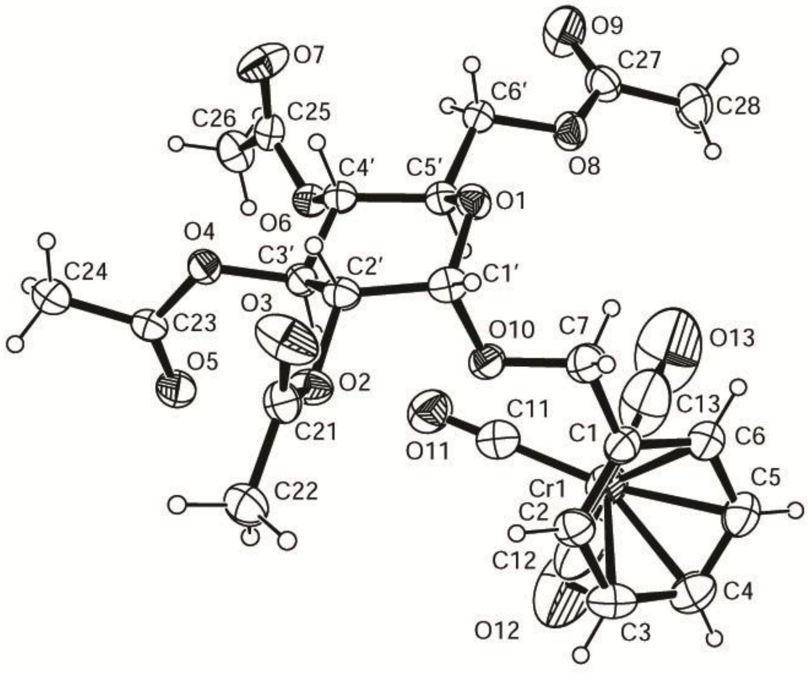

Figure S2: ORTEP-plot for compound **2b** showing 30% probability ellipsoids.

Table S2a: Atomic coordinates for compound **2b**.

	x	y	z	U(eq) ($pm^2 \cdot 10^{-1}$)
Xρ(1)	0.1022(1)	0.5224(1)	0.3620(1)	68(1)
O(1)	-0.3065(2)	0.6344(3)	0.1400(2)	44(1)
O(2)	-0.2135(2)	0.6053(3)	0.2950(2)	47(1)
O(3)	-0.4028(2)	0.4675(3)	0.3536(2)	51(1)
O(4)	-0.4719(2)	0.2247(3)	0.2110(2)	47(1)
O(5)	-0.3094(2)	0.1929(3)	0.0848(2)	43(1)
O(6)	-0.1457(2)	0.6131(4)	0.0066(2)	55(1)

O(7)	-0.5512(3)	0.6338(5)	0.3540(2)	81(1)
O(8)	-0.3738(2)	0.0422(4)	0.3104(2)	63(1)
O(9)	-0.4353(3)	0.1884(4)	-0.0499(2)	80(1)
O(10)	-0.1285(3)	0.5248(7)	-0.1374(3)	98(1)
O(11)	-0.0645(3)	0.2571(5)	0.2880(3)	82(1)
O(12)	0.2610(5)	0.2505(9)	0.4359(7)	209(4)
O(13)	0.1909(8)	0.512(2)	0.1794(6)	256(5)
X(1)	-0.0293(3)	0.7188(5)	0.3582(3)	53(1)
X(2)	-0.0239(4)	0.6281(6)	0.4423(3)	56(1)
X(3)	0.0794(4)	0.6097(7)	0.5036(4)	74(1)
X(4)	0.1787(5)	0.6790(8)	0.4807(5)	84(2)
X(5)	0.1757(5)	0.7693(8)	0.3988(4)	79(2)
X(6)	0.0712(4)	0.7880(7)	0.3376(4)	70(1)
X(7)	-0.1395(4)	0.7459(6)	0.2934(4)	61(1)
X(8)	-0.3191(3)	0.6290(5)	0.2376(3)	46(1)
X(9)	-0.4023(3)	0.4951(5)	0.2540(2)	43(1)
X(10)	-0.3734(3)	0.3305(5)	0.2128(3)	41(1)
X(11)	-0.3513(3)	0.3487(4)	0.1121(3)	39(1)
X(12)	-0.2646(3)	0.4852(4)	0.1058(3)	41(1)
X(13)	-0.2465(3)	0.5143(6)	0.0056(2)	45(1)
X(14)	-0.4841(3)	0.5398(6)	0.3944(3)	48(1)
X(15)	-0.4756(5)	0.4926(9)	0.4948(4)	63(1)
X(16)	-0.4605(3)	0.0831(4)	0.2611(3)	45(1)
X(17)	-0.5696(4)	-0.0125(6)	0.2450(3)	69(1)
X(18)	-0.3627(3)	0.1211(5)	0.0048(3)	48(1)
X(19)	-0.3199(5)	-0.0496(6)	-0.0052(4)	61(1)
X(20)	-0.0938(3)	0.6052(6)	-0.0696(3)	57(1)
X(21)	0.0107(4)	0.7108(7)	-0.0585(4)	78(1)
X(22)	-0.0010(4)	0.3611(7)	0.3164(4)	66(1)
X(23)	0.1989(5)	0.356(1)	0.4073(7)	121(3)
X(24)	0.1587(7)	0.513(1)	0.2512(6)	148(4)

Table S2b: Crystal data and structure refinement for **2b**.

Empirical formula	$C_{24}H_{26}CrO_{13}$
Formula weight	574.45
Temperature	220(2) K
Wavelength	71.073 pm
Crystal system	monoclinic
Space group	$P2_1$

Unit cell dimensions	*a* = 1176.7(2) pm	α = 90°
	b = 809.94(7) pm	β = 98.98(2)°
	c = 1423.6(2) pm	γ = 90°
Volume	1.3402(3) nm^3	
Z	2	
Density (calculated)	1.424 g/cm^3	
Absorption coefficient	0.492 mm^{-1}	
F(000)	596	
Crystal size	0,30 × 0,16 × 0,12 mm^3	
Theta range for data collection	2.90 to 28.13°.	
Index ranges	$-15 \le h \le 15$, $-10 \le k \le 10$, $-18 \le l \le 18$	
Reflections collected	15881	
Independent reflections	6505 [R_{int} = 0.0567]	
Completeness to theta = 28.13°	99.5%	
Absorption correction	None	
Refinement method	Full-matrix least-squares on F^2	
Data/restraints/parameters	6505/1/423	
Goodness-of-fit on F^2	0.898	
Final R indices [I>2sigma(I)]	R_1 = 0.0581, wR_2 = 0.1203	
R indices (all data)	R_1 = 0.0995, wR_2 = 0.1328	
Absolute structure parameter	-0.04(3)	
Largest diff. peak and hole	0.728 and -0.399 eÅ^{-3}	

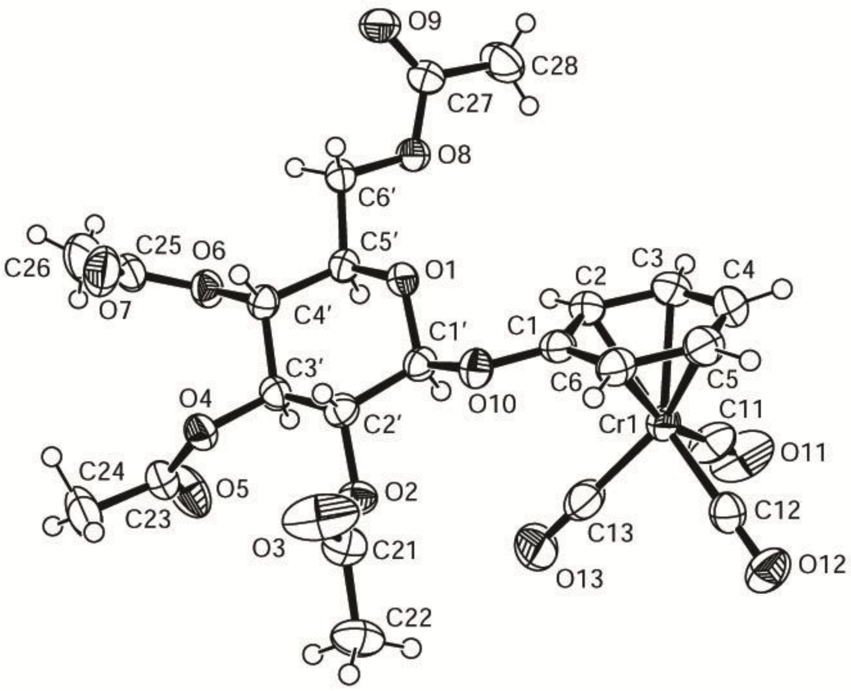

Figure S3: ORTEP-plot for compound **2c** showing 30% probability ellipsoids.

Table S3a: Atomic coordinates for compound **2c**.

	x	y	z	U(eq) (pm^{2}·10^{-1})
Cr(1)	0.9777(1)	0.8819(1)	0.5204(1)	34(1)
O(1)	1.13197(2)	1.1123(2)	0.1650(2)	33(1)
O(2)	1.0689(3)	1.1614(2)	0.3482(2)	34(1)
O(3)	0.7605(2)	1.2891(2)	0.1842(2)	35(1)
O(4)	0.9197(3)	1.4142(2)	-0.0464(2)	35(1)
O(5)	1.3284(3)	1.2333(2)	-0.1770(2)	37(1)
O(6)	1.7034(2)	0.9079(2)	0.0398(2)	36(1)
O(7)	0.6671(4)	1.5044(3)	0.2875(3)	77(1)
O(8)	0.8124(4)	1.3075(3)	-0.1881(2)	62(1)
O(9)	1.3118(4)	1.4701(3)	-0.1959(3)	60(1)
O(10)	2.0168(3)	0.9319(2)	0.0021(2)	45(1)
O(11)	0.9627(5)	0.5834(3)	0.4811(3)	83(1)
O(12)	0.6037(3)	0.9134(3)	0.7317(2)	52(1)
O(13)	0.6768(5)	1.0088(4)	0.3382(3)	83(1)
C(1)	1.1346(3)	1.0444(3)	0.4298(2)	33(1)
C(2)	1.0592(4)	1.0746(3)	0.5643(2)	37(1)
C(3)	1.1183(4)	0.9624(4)	0.6575(3)	45(1)
C(4)	1.2503(5)	0.8194(4)	0.6194(3)	48(1)
C(5)	1.3226(4)	0.7884(4)	0.4846(3)	45(1)
C(6)	1.2659(4)	0.8998(3)	0.3894(3)	36(1)
C(7)	1.1060(4)	1.1371(3)	0.2119(2)	31(1)
C(8)	0.9736(3)	1.2777(3)	0.1519(2)	31(1)
C(9)	1.0360(4)	1.2736(3)	0.0023(2)	31(1)
C(10)	1.2682(3)	1.2442(3)	-0.0367(2)	31(1)
C(11)	1.3830(3)	1.0974(3)	0.0248(2)	30(1)
C(12)	1.6156(4)	1.0589(3)	-0.0024(2)	32(1)
C(13)	0.6212(4)	1.4084(3)	0.2510(3)	42(1)
C(14)	0.4052(4)	1.4067(4)	0.2649(3)	52(1)
C(15)	0.8152(4)	1.4171(3)	-0.1436(3)	42(1)
C(16)	0.7110(7)	1.5719(5)	-0.1843(6)	67(1)
C(17)	1.3487(4)	1.3533(4)	-0.2447(3)	45(1)
C(18)	14192(8)	1.3214(7)	-0.3886(4)	72(1)
C(19)	19076(4)	0.8583(3)	0.0397(3)	35(1)
C(20)	19821(6)	0.7047(4)	0.0922(5)	58(1)
C(21)	0.9719(5)	0.6969(4)	0.4949(3)	53(1)
C(22)	0.7476(4)	0.9023(3)	0.6522(3)	38(1)
C(23)	0.7926(5)	0.9590(4)	0.4073(3)	54(1)

Table S3b: Crystal data and structure refinement for **2c**.

Empirical formula	$C_{23}H_{24}CrO_{13}$
Formula weight	560.42
Temperature	210(2) K
Wavelength	71.073 pm
Crystal system	Triclinic
Space group	*P*1

Unit cell dimensions	*a* = 700.94(7) pm	α =85.20(1)°.
	b = 969.2(1) pm	β = 78.36(1)°.
	c = 1043.54(9) pm	χ = 69.23(1)°.
Volume	0.64914(11) nm^3	
Z	1	
Density (calculated)	1.434 g/cm^3	
Absorption coefficient	0.506 mm^{-1}	
F(000)	290	
Crystal size	0.62 × 0.19 × 0.03 mm^3	
Theta range for data collection	3.42 to 30.22°.	
Index ranges	-9<=h<=9, -13<=k<=13, -14<=l<=14	
Reflections collected	12319	
Independent reflections	7011 [R(int) = 0.0484]	
Completeness to theta = 30.22°	91.2%	
Refinement method	Full-matrix least-squares on F^2	
Data/restraints/parameters	7011/3/430	
Goodness-of-fit on F^2	0.910	
Final R indices [I>2sigma(I)]	R1 = 0.0413, wR2 = 0.0900	
R indices (all data)	R1 = 0.0561, wR2 = 0.0951	
Absolute structure parameter	-0.005(14)	
Largest diff. peak and hole	0.412 and -0.257 eÅ^{-3}	

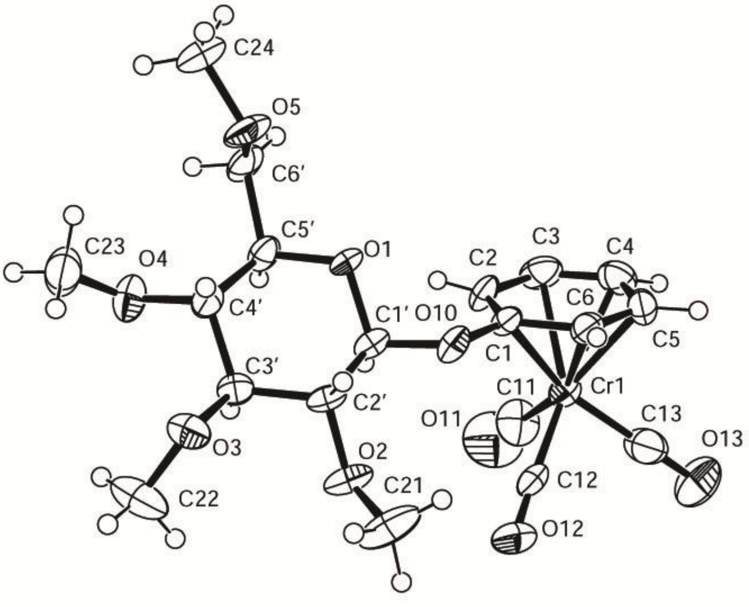

Figure S4: ORTEP-plot for compound **2d** showing 30% probability ellipsoids.

Table S4a: Atomic coordinates for compound **2d**.

	x	y	z	U(eq) ($pm^2 \cdot 10^{-1}$)
Cr(1)	0.7766(1)	0.6142(1)	0.1285(1)	33(1)
O(1)	0.4276(3)	0.8799(4)	0.2544(3)	33(1)
O(2)	0.6374(3)	0.8874(4)	0.2501(3)	34(1)
O(3)	0.7231(4)	0.8228(5)	0.4841(4)	46(1)
O(4)	0.5363(4)	0.9398(4)	0.5954(3)	40(1)
O(5)	0.2809(4)	0.8085(5)	0.4802(4)	48(1)
O(6)	0.1918(4)	1.0426(5)	0.2394(4)	51(1)
O(7)	0.9399(4)	0.6694(6)	0.3648(4)	58(1)
O(8)	0.7500(8)	0.2676(6)	0.1759(7)	100(2)
O(9)	1.0289(6)	0.5272(8)	0.0835(6)	89(2)
C(1)	0.5568(4)	0.8270(6)	0.3112(4)	30(1)
C(2)	0.6022(5)	0.8958(6)	0.4293(5)	31(1)
C(3)	0.5057(6)	0.8526(7)	0.4927(5)	30(1)
C(4)	0.3647(5)	0.8869(6)	0.4275(5)	32(1)
C(5)	0.3348(5)	0.8224(6)	0.3083(5)	34(1)
C(6)	0.1993(5)	0.8757(8)	0.2354(6)	44(1)
C(7)	0.8314(6)	0.9279(9)	0.5192(8)	71(2)
C(8)	0.5715(9)	0.8472(9)	0.6936(5)	65(2)
C(9)	0.2079(7)	0.9078(9)	0.5301(6)	57(2)
C(10)	0.0610(5)	1.102(1)	0.2122(6)	70(2)
C(11)	0.6422(4)	0.8107(6)	0.1534(4)	28(1)
C(12)	0.5654(5)	0.6768(7)	0.1060(5)	37(1)
C(13)	0.5799(5)	0.612(1)	0.0057(5)	49(1)
C(14)	0.6663(7)	0.6760(8)	-0.0465(5)	46(1)
C(15)	0.7448(6)	0.8067(7)	0.0034(5)	44(1)
C(16)	0.7320(5)	0.8724(7)	0.1016(5)	34(1)
C(17)	0.8752(5)	0.6465(7)	0.2744(5)	39(1)
C(18)	0.7594(8)	0.3981(7)	0.1602(7)	58(2)
C(19)	0.9291(7)	0.5618(8)	0.1009(6)	56(2)

Table S4b: Crystal data and structure refinement for **2d**.

Empirical formula	$C_{19}H_{24}CrO_9$	
Formula weight	448.38	
Temperature	220(2) K	
Wavelength	71.073 pm	
Crystal system	Monoclinic	
Space group	$P2_1$	
Unit cell dimensions	a = 1067.5(1) pm	α = 90°
	b = 833.6(1) pm	β = 106.64(1)°
	c = 1243.9(1) pm	γ = 90°
Volume	1.0605(2) nm^3	
Z	2	
Density (calculated)	1.404 g/cm^3	
Absorption coefficient	0.586 mm^{-1}	

F(000)	468
Crystal size	1.10 × 1.00 × 0.20 mm^3
Theta range for data collection	2.97 to 25.97°.
Index ranges	-13 ≤ h ≤ 13, -10 ≤ k ≤ 10, -15 ≤ l ≤ 15
Reflections collected	12669
Independent reflections	4111 [R(int) = 0.2126]
Completeness to theta = 25.97°	98.8%
Absorption correction	None
Max. and min. transmission	0.8918 and 0.5651
Refinement method	Full-matrix least-squares on F^2
Data/restraints/parameters	4111/1/310
Goodness-of-fit on F^2	0.970
Final R indices [I>2sigma(I)]	R_1 = 0.0806, wR_2 = 0.1739
R indices (all data)	R_1 = 0.0890, wR_2 = 0.1795
Absolute structure parameter	-0.05(4)
Largest diff. peak and hole	1.611 and -1.520 eÅ^{-3}

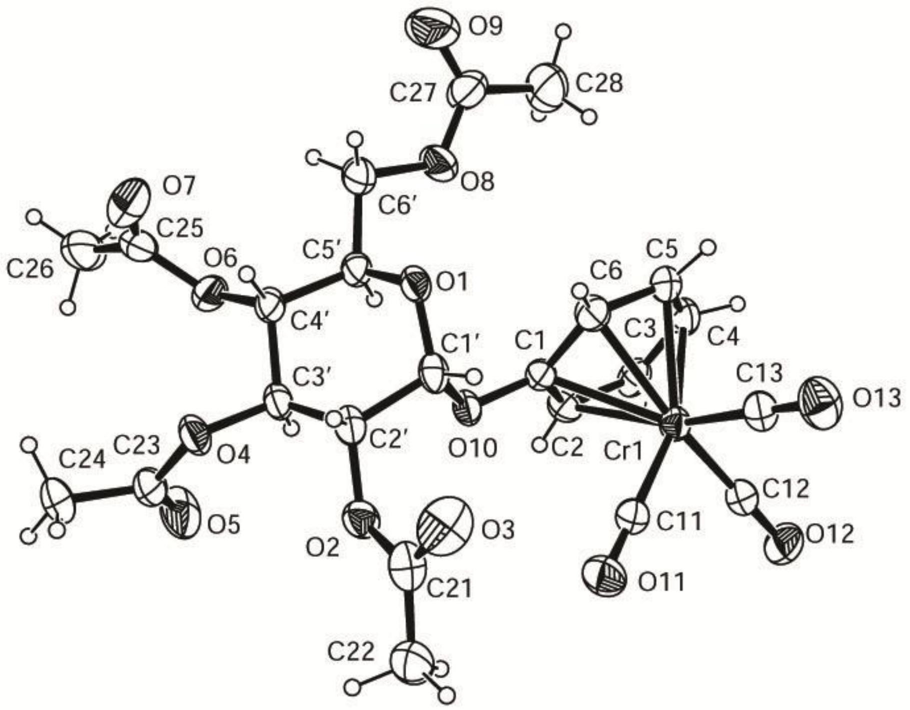

Figure S5: ORTEP-plot for compound **2e** showing 30% probability ellipsoids.

Table S5a:. Atomic coordinates for compound **2e**.

	x	y	z	U(eq) ($pm^2 \cdot 10^{-1}$)
Cr(1)	0.3167(1)	0.2061(1)	0.0352(1)	46(1)
O(1)	0.1846(3)	0.2710(4)	0.1594(1)	50(1)
O(2)	0.0927(3)	0.2644(4)	0.1013(1)	48(1)
O(3)	-0.1118(3)	0.0209(4)	0.1202(1)	58(1)
O(4)	-0.2225(3)	0.1914(4)	0.1831(1)	59(1)
O(5)	-0.1027(3)	0.5366(4)	0.1970(1)	68(1)
O(6)	0.3036(4)	0.6319(5)	0.1615(1)	77(1)
O(7)	0.0215(5)	-0.2296(5)	0.1192(1)	100(1)
O(8)	-0.3754(3)	0.3384(7)	0.1481(1)	103(2)
O(9)	-0.0683(5)	0.4827(7)	0.2555(1)	110(2)
O(10)	0.4473(5)	0.718(1)	0.2024(2)	175(3)
O(11)	0.1028(4)	-0.0924(5)	0.0373(1)	75(1)
O(12)	0.2980(4)	0.1926(5)	-0.0450(1)	80(1)
O(13)	0.5368(4)	-0.0848(5)	0.0315(1)	85(1)
C(1)	0.2060(5)	0.3289(6)	0.0830(1)	44(1)
C(2)	0.1793(7)	0.4350(6)	0.0525(1)	52(1)
C(3)	0.2854(6)	0.5083(6)	0.0321(1)	57(1)
C(4)	0.4240(6)	0.4758(7)	0.0419(1)	60(1)
C(5)	0.4524(5)	0.3682(7)	0.0714(1)	57(1)
C(6)	0.3438(5)	0.2935(7)	0.0922(1)	55(1)
C(7)	0.1159(5)	0.1655(7)	0.1335(1)	48(1)
C(8)	-0.0255(5)	0.1016(8)	0.1476(1)	49(1)
C(9)	-0.1067(5)	0.2619(7)	0.1627(1)	49(1)
C(10)	-0.0196(5)	0.3791(7)	0.1874(1)	50(1)
C(11)	0.1096(5)	0.4383(7)	0.1676(1)	51(1)
C(12)	0.2062(6)	0.5653(9)	0.1878(1)	63(1)
C(13)	-0.0819(7)	-0.1521(9)	0.1084(2)	72(2)
C(14)	-0.1794(5)	-0.2190(7)	0.0811(2)	89(2)
C(15)	-0.3519(5)	0.2507(8)	0.1743(2)	69(2)
C(16)	-0.4529(5)	0.195(1)	0.2026(1)	91(2)
C(17)	-0.1277(5)	0.5670(9)	0.2327(2)	75(2)
C(18)	-0.2276(6)	0.718(1)	0.2372(2)	122(2)
C(19)	0.4157(7)	0.7094(9)	0.1712(2)	88(2)
C(20)	0.5058(6)	0.7722(8)	0.1413(2)	106(2)
C(21)	0.1853(5)	0.0222(7)	0.0364(1)	52(1)
C(22)	0.3056(4)	0.1984(6)	-0.0136(1)	54(1)
C(23)	0.4501(5)	0.0302(7)	0.0333(1)	56(1)

Table S5b: Crystal data and structure refinement for **2e**.

Empirical formula	$C_{23}H_{24}CrO_{13}$
Formula weight	560.42
Temperature	293(2) K
Wavelength	71.073 pm
Crystal system	Orthorhombic
Space group	$P2_12_12_1$

Unit cell dimensions	*a* = 962.95(9) pm	α = 90°
	b = 719.12(6) pm	β = 90°
	c = 3703.7(4) pm	γ = 90°
Volume	2.5647(4) nm^3	
Z	4	
Density (calculated)	1.451 g/cm^3	
Absorption coefficient	0.512 mm^{-1}	
F(000)	1160	
Crystal size	0.16 × 0.26 × 0.38 mm^3	
Theta range for data collection	2.19 to 19.55°.	
Index ranges	$-9 \le h \le 9$, $-6 \le k \le 6$, $-34 \le l \le 34$	
Reflections collected	9977	
Independent reflections	2246 [R_{int} = 0.0388]	
Completeness to theta = 19.55°	99.7%	
Refinement method	Full-matrix least-squares on F^2	
Data/restraints/parameters	2246/0/370	
Goodness-of-fit on F^2	1.003	
Final R indices [I>2sigma(I)]	R_1 = 0.0291, wR_2 = 0.0644	
R indices (all data)	R_1 = 0.0346, wR_2 = 0.0658	
Absolute structure parameter	-0.01(3)	
Largest diff. peak and hole	0.229 and -0.155 $eÅ^3$	

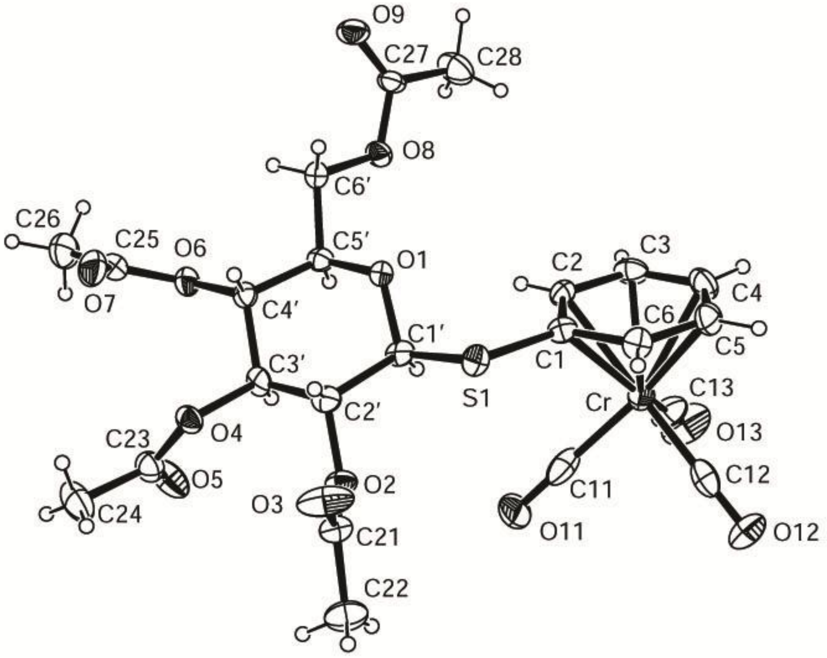

Figure S6: ORTEP-plot for compound **2j** showing 30% probability ellipsoids.

Table S6a: Atomic coordinates for compound **2j**.

	x	y	z	U(eq) ($pm^2 \cdot 10^{-1}$)
Cr(1)	0.1621(1)	0.2390(1)	-1.7414(1)	41(1)
S(1)	0.2167(2)	0.5755(2)	-1.8753(1)	38(1)
O(1)	0.4803(5)	0.5055(4)	-2.0967(3)	34(1)
O(2)	-0.0731(5)	0.6810(4)	-2.0893(3)	38(1)
O(3)	-0.1521(7)	0.8857(6)	-1.9868(5)	75(2)
O(4)	0.1011(5)	0.7855(4)	-2.3243(3)	37(1)
O(5)	-0.0212(7)	0.6778(6)	-2.4574(4)	71(1)
O(6)	0.5035(6)	0.5970(5)	-2.4471(3)	40(1)
O(7)	0.4959(7)	0.8237(6)	-2.4785(5)	63(1)
O(8)	0.8585(5)	0.2937(4)	-2.2220(3)	39(1)
O(9)	1.1708(6)	0.3135(5)	-2.2492(4)	50(1)
O(10)	0.1352(9)	-0.0435(6)	-1.7955(6)	95(2)
O(11)	-0.2131(7)	0.2739(5)	-1.5303(4)	61(1)
O(12)	-0.1308(8)	0.3741(7)	-1.9286(5)	82(2)
C(1)	0.2667(9)	0.5372(7)	-2.0474(6)	37(2)
C(2)	0.1422(9)	0.6636(8)	-2.1209(6)	36(2)
C(3)	0.2092(9)	0.6531(7)	-2.2668(6)	34(1)
C(4)	.4412(8)	0.6169(6)	-2.3086(5)	30(1)
C(5)	0.5455(8)	0.4836(7)	-2.2354(5)	33(1)
C(6)	0.7764(9)	0.4393(7)	-2.2626(6)	33(1)
C(7)	-0.2074(9)	0.7989(8)	-2.0275(5)	41(2)
C(8)	-0.423(1)	0.801(1)	-2.0163(9)	57(2)
C(9)	-0.0070(9)	0.7843(7)	-2.4186(6)	46(2)
C(10)	-0.107(2)	0.931(1)	-2.468(1)	72(2)
C(11)	0.5317(9)	0.7068(9)	-2.5229(6)	46(2)
C(12)	0.607(1)	0.6680(9)	-2.6639(6)	72(2)
C(13)	1.0569(8)	0.2440(7)	-2.2175(5)	38(1)
C(14)	1.125(1)	0.0929(8)	-2.1721(7)	67(2)
C(15)	0.3088(8)	0.4086(6)	-1.8002(5)	34(1)
C(16)	0.4368(8)	0.2809(6)	-1.8662(6)	36(2)
C(17)	0.5054(9)	0.1545(8)	-1.7961(7)	46(2)
C(18)	0.450(1)	0.1523(9)	-1.6579(7)	51(2)
C(19)	0.328(1)	0.2748(8)	-1.5917(6)	50(2)
C(20)	0.255(1)	0.4036(8)	-1.6617(6)	40(2)
C(21)	0.150(1)	0.0663(9)	-1.7741(7)	59(2)
C(22)	-0.068(1)	0.2616(7)	-1.6122(6)	47(2)
C(23)	-0.017(1)	0.3213(9)	-1.8568(6)	61(2)

Table S6b: Crystal data and structure refinement for **2j**.

Empirical formula	$C_{23}H_{24}CrO_{12}S$	
Formula weight	576.48	
Temperature	220(2) K	
Wavelength	71.073 pm	
Crystal system	Triclinic	
Space group	*P*1	
Unit cell dimensions	*a* = 695.6(2) pm	α = 85.96(2)°
	b = 997.0(2) pm	β = 78.98(3)°
	c = 1027.8(2) pm	γ = 70.31(2)°
Volume	0.6588(2) nm^3	
Z	1	
Density (calculated)	1.453 g/cm^3	
Absorption coefficient	0.574 mm^{-1}	
F(000)	298	
Crystal size	0.26 × 0.05 × 0.04 mm^3	
Theta range for data collection	2.95 to 26.03°	
Index ranges	-8 ≤ h ≤ 8, -12 ≤ k ≤ 12, -12 ≤ l ≤ 12	
Reflections collected	6587	
Independent reflections	4792 [R_{int} = 0.0624]	
Completeness to theta = 26.03°	93.3%	
Absorption correction	None	
Max. and min. transmission	0.9774 and 0.8651	
Refinement method	Full-matrix least-squares on F^2	
Data/restraints/parameters	4792/3/406	
Goodness-of-fit on F^2	0.829	
Final R indices [I>2sigma(I)]	R_1 = 0.0496, wR_2 = 0.0806	
R indices (all data)	R_1 = 0.0898, wR_2 = 0.0887	
Absolute structure parameter	-0.02(3)	
Largest diff. peak and hole	0.545 and -0.228 eÅ^{-3}	

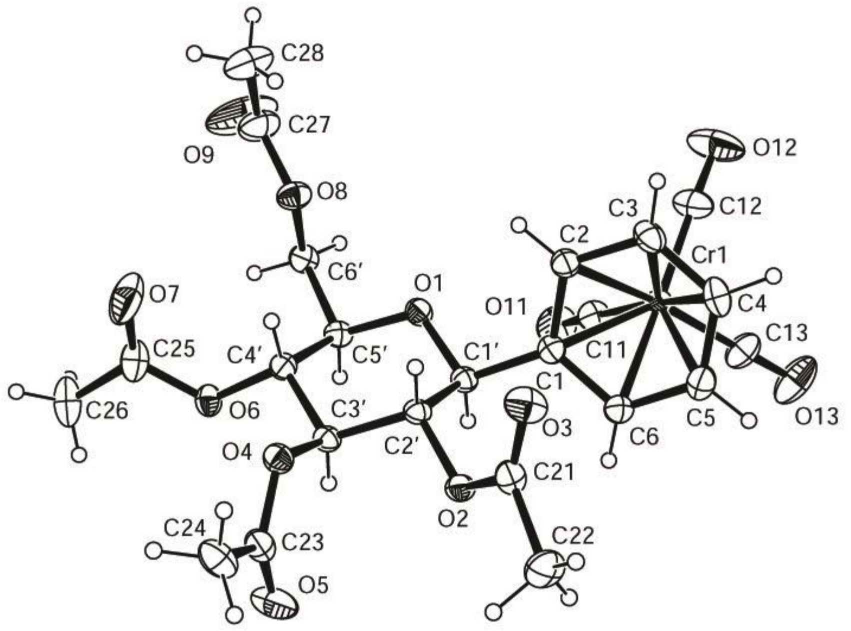

Figure S7: ORTEP-plot for compound **2k** showing 30% probability ellipsoids.

Table S7a: Atomic coordinates for compound **2k**.

	x	y	z	U(eq) (pm$^2 \cdot 10^{-1}$)
Cr(1)	0.5006(1)	0.6908(1)	0.1194(1)	33(1)
O(1)	0.7693(2)	0.8949(1)	0.1702(1)	34(1)
O(2)	0.4995(2)	1.0275(1)	0.0670(1)	34(1)
O(3)	0.6674(2)	1.1665(1)	0.1322(1)	35(1)
O(4)	0.9869(2)	1.1114(1)	0.1573(1)	37(1)
O(5)	0.9182(2)	0.9466(1)	0.2853(1)	43(1)
O(6)	0.2905(2)	1.0076(1)	0.1322(1)	52(1)
O(7)	0.7054(3)	1.2135(1)	0.0272(1)	67(1)
O(8)	0.9882(4)	1.1671(2)	0.2607(1)	88(1)
O(9)	1.1151(4)	0.9025(4)	0.3469(2)	134(2)
O(10)	0.5230(5)	0.5301(2)	0.2118(1)	98(1)
O(11)	0.8432(3)	0.6774(2)	0.0938(1)	71(1)
O(12)	0.4392(3)	0.5560(2)	0.0111(1)	83(1)
C(1)	0.5288(2)	0.8431(1)	0.1243(1)	32(1)
C(2)	0.4826(3)	0.8122(2)	0.1876(1)	36(1)
C(3)	0.3456(3)	0.7578(2)	0.1932(1)	43(1)
C(4)	0.2565(3)	0.7365(2)	0.1378(2)	46(1)
C(5)	0.3044(3)	0.7665(2)	0.0753(1)	43(1)
C(6)	0.4399(3)	0.8198(2)	0.0676(1)	37(1)
C(7)	0.6640(2)	0.9088(1)	0.1172(1)	30(1)
C(8)	0.6013(2)	1.0078(1)	0.1217(1)	29(1)
C(9)	0.7325(2)	1.0769(1)	0.1184(1)	30(1)
C(10)	0.8527(2)	1.0552(2)	0.1711(1)	31(1)
C(11)	0.9025(3)	0.9536(2)	0.1678(1)	32(1)
C(12)	1.0049(3)	0.9260(2)	0.2259(1)	37(1)

C(13)	0.3446(3)	1.0288(2)	0.0803(1)	38(1)
C(14)	0.2573(4)	1.0607(3)	0.0205(2)	57(1)
C(15)	0.6523(3)	1.2263(2)	0.0814(1)	40(1)
C(16)	0.5613(4)	1.3088(2)	0.1025(2)	51(1)
C(17)	1.0445(3)	1.1639(2)	0.2070(2)	49(1)
C(18)	1.1874(4)	1.2141(2)	0.1851(3)	64(1)
C(19)	0.9885(4)	0.9343(2)	0.3430(1)	63(1)
C(20)	0.8867(6)	0.9621(4)	0.3997(2)	77(1)
C(21)	0.5161(5)	0.5922(2)	0.1771(1)	54(1)
C(22)	0.7131(3)	0.6824(2)	0.1047(1)	45(1)
C(23)	0.4668(3)	0.6071(2)	0.0525(1)	51(1)

Table S7b: Crystal data and structure refinement for **2k**.

Empirical formula	$C_{23}H_{24}CrO_{12}$	
Formula weight	544.42	
Temperature	220(2) K	
Wavelength	71.073 pm	
Crystal system	Orthorhombic	
Space group	$P\ 2_1\ 2_1\ 2_1$	
Unit cell dimensions	a = 856.97(9) pm	α = 90°.
	b = 1449.0(2) pm	β = 90°.
	c = 2008.5(2) pm	γ = 90°.
Volume	2.4941(5) nm^3	
Z	4	
Density (calculated)	1.450 g/cm^3	
Absorption coefficient	0.521 mm^{-1}	
F(000)	1128	
Crystal size	0.44 × 0.22 × 0.10 mm^3	
Theta range for data collection	2.76 to 28.11°.	
Index ranges	$-11 \le h \le 11$, $-19 \le k \le 19$, $-26 \le l \le 26$	
Reflections collected	33174	
Independent reflections	6047 [R_{int} = 0.0800]	
Completeness to theta = 28.11°	99.4%	
Absorption correction	None	
Max. and min. transmission	0.9497 and 0.8031	
Refinement method	Full-matrix least-squares on F^2	
Data/restraints/parameters	6047/0/421	
Goodness-of-fit on F^2	0.968	
Final R indices [I>2sigma(I)]	R_1 = 0.0397, wR_2 = 0.0863	
R indices (all data)	R_1 = 0.0500, wR_2 = 0.0895	
Absolute structure parameter	-0.008(18)	
Largest diff. peak and hole	0.515 and -0.199 eÅ^{-3}	

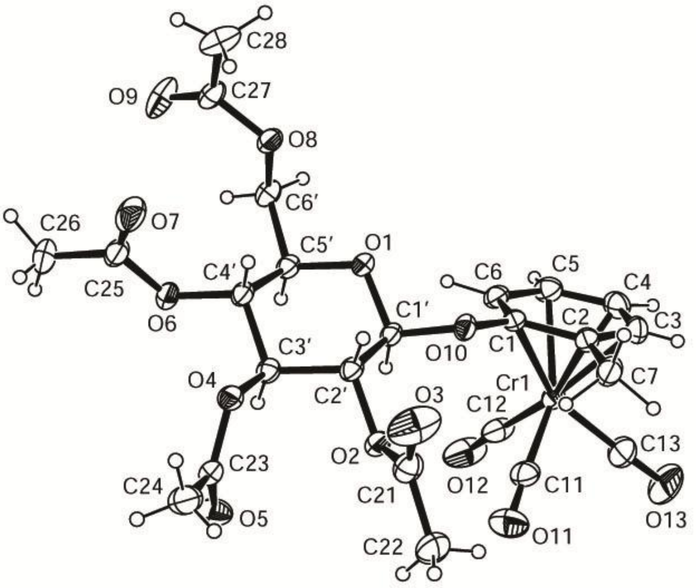

Figure S8: ORTEP-plot for compound ***pR*-2m** showing 30% probability ellipsoids.

Table S8a: Atomic coordinates for compound ***pR*-2m**.

	x	y	z	U(eq) ($pm^2 \cdot 10^{-1}$)
Cr(1)	0.9885(1)	1.0278(1)	0.7604(1)	37(1)
O(1)	1.1524(1)	1.0072(2)	0.6681(1)	34(1)
O(2)	1.0729(1)	0.8645(2)	0.6593(1)	34(1)
O(3)	1.1533(1)	0.6714(2)	0.8285(1)	37(1)
O(4)	1.2701(1)	0.6562(2)	0.8406(1)	35(1)
O(5)	1.3171(1)	0.9575(2)	0.8226(2)	41(1)
O(6)	1.2246(1)	1.1053(2)	0.5663(1)	39(1)
O(7)	1.1303(2)	0.4704(3)	0.7140(3)	91(1)
O(8)	1.2881(1)	0.6388(3)	1.0182(2)	52(1)
O(9)	1.3408(1)	0.8638(3)	0.6938(2)	60(1)
O(10)	1.3128(1)	1.2150(3)	0.5984(2)	68(1)
O(11)	1.0420(1)	0.7497(3)	0.9001(2)	76(1)
O(12)	0.8880(1)	1.0025(6)	0.8197(3)	124(2)
O(13)	1.0586(2)	1.2095(4)	0.9677(2)	94(1)
C(1)	1.1331(1)	0.9091(3)	0.7291(2)	31(1)
C(2)	1.1704(1)	0.7624(3)	0.7605(2)	32(1)
C(3)	1.2379(1)	0.7990(3)	0.8249(2)	32(1)
C(4)	1.2550(1)	0.9098(3)	0.7584(2)	33(1)
C(5)	1.2148(1)	1.0526(3)	0.7313(2)	34(1)
C(6)	1.2275(1)	1.1733(3)	0.6646(2)	40(1)

C(7)	1.1339(1)	0.5259(4)	0.7965(2)	47(1)
C(8)	1.1181(2)	0.4505(5)	0.8765(4)	62(1)
C(9)	1.2891(1)	0.5824(3)	0.9390(2)	36(1)
C(10)	1.3093(2)	0.4229(4)	0.9336(3)	52(1)
C(11)	1.3556(1)	0.9302(3)	0.7791(3)	45(1)
C(12)	1.4163(1)	1.0007(6)	0.8507(4)	69(1)
C(13)	1.2706(1)	1.1349(3)	0.5424(2)	41(1)
C(14)	1.2615(2)	1.0540(5)	0.4401(4)	65(1)
C(15)	1.0266(1)	0.9619(3)	0.6454(2)	33(1)
C(16)	1.0337(1)	1.1201(3)	0.6638(2)	39(1)
C(17)	0.9829(1)	1.2124(4)	0.6432(3)	46(1)
C(18)	0.9257(1)	1.1463(4)	0.6046(2)	50(1)
C(19)	0.9194(1)	0.9852(4)	0.5881(2)	45(1)
C(20)	0.9689(1)	0.8902(3)	0.6095(2)	38(1)
C(21)	0.9613(1)	0.7188(4)	0.5948(3)	46(1)
C(22)	1.0224(1)	0.8573(4)	0.8475(2)	49(1)
C(23)	0.9272(1)	1.0121(6)	0.7992(3)	67(1)
C(24)	1.0315(2)	1.1439(4)	0.8864(3)	57(1)

Table S8b: Crystal data and structure refinement for ***pR*-2m**.

Empirical formula	$C_{24}H_{26}CrO_{13}$	
Formula weight	574.45	
Temperature	220(2) K	
Wavelength	71.073 pm	
Crystal system	Monoclinic	
Space group	*C*2	
Unit cell dimensions	*a* = 2479.8(4) pm	α = 90°
	b = 862.75(8) pm	β = 117.66(2)°
	c = 1360.2(2) pm	γ = 90°
Volume	2.5774(6) nm^3	
Z	4	
Density (calculated)	1.480 g/cm^3	
Absorption coefficient	0.511 mm^{-1}	
F(000)	1192	
Crystal size	0.28 × 0.20 × 0.18 mm^3	
Theta range for data collection	2.54 to 26.00°	
Index ranges	-30 ≤ h ≤ 30, -10 ≤ k ≤ 10, -16 ≤ l ≤ 16	
Reflections collected	15530	
Independent reflections	5048 [R_{int} = 0.0332]	
Completeness to theta = 26.00°	99.5%	
Absorption correction	None	
Refinement method	Full-matrix least-	

	squares on F^2
Data/restraints/parameters	5048/1/447
Goodness-of-fit on F^2	0.952
Final R indices [I>2sigma(I)]	R_1 = 0.0326, wR_2 = 0.0718
R indices (all data)	R_1 = 0.0400, wR_2 = 0.0747
Absolute structure parameter	-0.01(1)
Largest diff. peak and hole	0.420 and -0.206 eÅ^{-3}

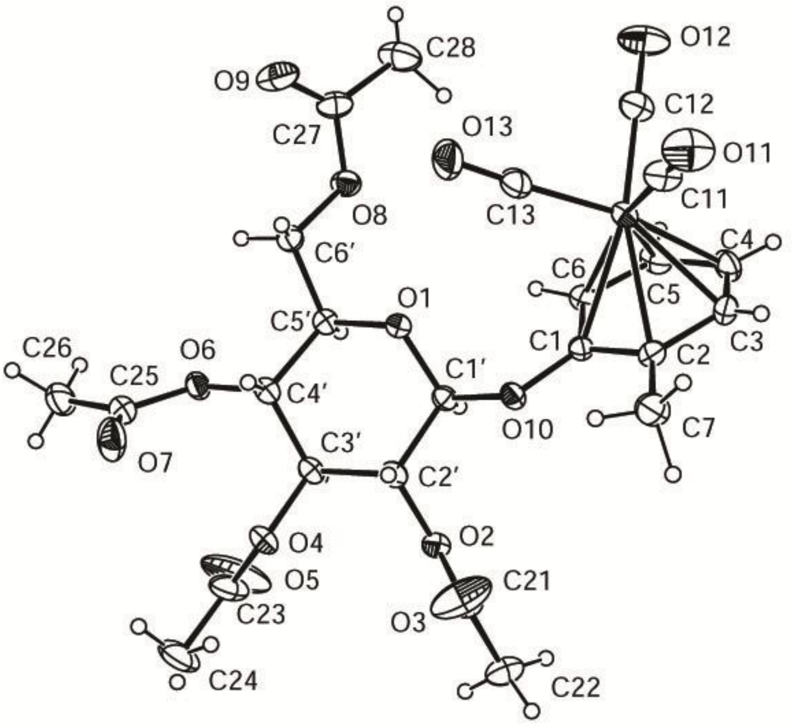

Figure S9: ORTEP-plot for compound ***pS*-2m** showing 30% probability ellipsoids.

Table S9a: Atomic coordinates for compound ***pS*-2m**.

	x	y	z	U(eq) (pm^2·10^{-1})
Cr(1)	-0.0071(1)	-0.0756(1)	0.3063(1)	31(1)
O(1)	-0.1028(3)	-0.3081(2)	-0.0355(2)	30(1)
O(2)	0.1175(3)	-0.3344(2)	0.1461(2)	29(1)
O(3)	0.3120(3)	-0.4908(2)	-0.0073(2)	31(1)
O(4)	0.0139(4)	-0.6440(2)	-0.2156(2)	36(1)
O(5)	-0.2322(4)	-0.4663(2)	-0.3629(2)	35(1)

O(6)	-0.3065(4)	-0.1193(2)	-0.1648(2)	38(1)
O(7)	0.2357(5)	-0.6534(4)	0.1314(4)	88(1)
O(8)	0.195(1)	-0.5848(4)	-0.3698(4)	121(2)
O(9)	-0.4130(5)	-0.6945(3)	-0.3767(3)	62(1)
O(10)	-0.6481(5)	-0.1285(3)	-0.1977(3)	61(1)
O(11)	-0.2452(6)	-0.1648(4)	0.5143(3)	73(1)
O(12)	-0.4052(5)	-0.2059(4)	0.1164(3)	74(1)
O(13)	-0.1682(6)	0.1830(3)	0.2974(4)	79(1)
C(1)	0.0956(5)	-0.3328(3)	0.0118(3)	26(1)
C(2)	0.1007(5)	-0.4782(3)	-0.0372(3)	29(1)
C(3)	0.0285(6)	-0.4980(3)	-0.1828(3)	30(1)
C(4)	-0.1798(5)	-0.4634(3)	-0.2257(3)	31(1)
C(5)	-0.1578(5)	-0.3127(3)	-0.1728(3)	28(1)
C(6)	-0.3586(5)	-0.2677(3)	-0.2047(3)	33(1)
C(7)	0.3590(6)	-0.5846(4)	0.0762(4)	42(1)
C(8)	0.5826(7)	-0.5841(5)	0.0918(5)	49(1)
C(9)	0.1054(7)	-0.6738(4)	-0.3116(4)	55(1)
C(10)	0.067(1)	-0.8296(6)	-0.3350(7)	71(2)
C(11)	-0.3568(6)	-0.5901(4)	-0.4283(4)	42(1)
C(12)	-0.3987(9)	-0.5737(5)	-0.5684(4)	56(1)
C(13)	-0.4670(6)	-0.0640(4)	-0.1618(3)	42(1)
C(14)	-0.393(1)	0.0867(5)	-0.1070(6)	67(1)
C(15)	0.1765(4)	-0.2092(3)	0.2196(3)	28(1)
C(16)	0.2039(5)	-0.0753(3)	0.1688(3)	32(1)
C(17)	0.2827(5)	0.0468(3)	0.2534(4)	41(1)
C(18)	0.3260(6)	0.0365(4)	0.3864(4)	44(1)
C(19)	0.2818(6)	-0.0959(4)	0.4343(4)	40(1)
C(20)	0.2080(5)	-0.2220(3)	0.3540(3)	34(1)
C(21)	0.1755(8)	-0.3632(4)	0.4100(4)	49(1)
C(22)	-0.1542(6)	-0.1311(4)	0.4346(4)	47(1)
C(23)	-0.2492(6)	-0.1585(4)	0.1888(4)	47(1)
C(24)	-0.1056(7)	0.0847(4)	0.3005(4)	47(1)

Table S9b: Crystal data and structure refinement for ***pS*-2m**.

Empirical formula	$C_{24}H_{26}CrO_{13}$
Formula weight	574.45
Temperature	220(2) K
Wavelength	71.073 pm
Crystal system	Triclinic
Space group	*P*1

Unit cell dimensions	*a* = 667.4(1) pm	α = 91.08(2)°
	b = 977.3(1) pm	β = 100.89(2)°
	c = 1059.3(2) pm	γ = 104.35(2)°
Volume	0.65572(17) nm^3	
Z	1	
Density (calculated)	1.455 g/cm^3	
Absorption coefficient	0.502 mm^{-1}	
F(000)	298	
Crystal size	0.60 × 0.14 × 0.12 mm^3	
Theta range for data collection	2.81 to 25.95°	
Index ranges	$-8 \le h \le 8$, $-12 \le k \le 11$, $-12 \le l \le 12$	
Reflections collected	7846	
Independent reflections	4766 [R_{int} = 0.0448]	
Completeness to theta = 25.95°	93.6%	
Absorption correction	None	
Refinement method	Full-matrix least-squares on F^2	
Data/restraints/parameters	4766/3/447	
Goodness-of-fit on F^2	1.022	
Final R indices [I>2sigma(I)]	R_1 = 0.0407, wR_2 = 0.0947	
R indices (all data)	R_1 = 0.0432, wR_2 = 0.0957	
Absolute structure parameter	-0.01(2)	
Largest diff. peak and hole	0.498 and -0.235 eÅ^{-3}	

References

1. Sheldrick, G. M. *Acta Crystallogr., Sect. A* **2008,** *A64,* 112–122. doi:10.1107/S0108767307043930
2. Spek, A. L. *J. Appl. Crystallogr.* **2003,** *36,* 7–13. doi:10.1107/S0021889802022112
3. Barnes, C. L. *J. Appl. Crystallogr.* **1997,** *30,* 568. doi:10.1107/S0021889897006638
4. Farrugia, L. J. *J. Appl. Crystallogr.* **1997,** *30,* 565. doi:10.1107/S0021889897003117
5. Steffen, D.; Vogel, C.; Kristen, H. *Carbohydr. Res.* **1990,** *204,* 109–120. doi:10.1016/0008-6215(90)84026-Q
6. Utamura, T.; Kuromatsu, K.; Suwa, K.; Koizumi, K.; Shingu, T. *Chem. Pharm. Bull.* **1986,** *34,* 2341–2353. doi:10.1248/cpb.34.2341
7. Dess, D.; Kleine, H. P.; Weinberg, D. V.; Kaufmann, R. J.; Sidhu, R. S. *Synthesis* **1981,** 883–885. doi:10.1055/s-1981-29631

8. Stanssens, D.; De Keukeleire, D.; Vandewalle, M. *Tetrahedron: Asymmetry* **1990,** *1,* 547–560. doi:10.1016/S0957-4166(00)80546-7
9. Audichya, T. D.; Ingle, T. R.; Bose, J. L. *Indian J. Chem.* **1973,** *11,* 704–705.
10. Montgomery, E. M.; Richtmyer, N. K.; Hudson, C. S. *J. Am. Chem. Soc.* **1942,** *64,* 690–694. doi:10.1021/ja01255a060
11. Pfander, H.; Läderach, M. *Carbohydr. Res.* **1982,** *99,* 175–179. doi:10.1016/S0008-6215(00)81907-2
12. Baker, J. W. *J. Chem. Soc.* **1928,** 1583–1593.
13. Dasgupta, F.; Garegg, P. J. *Acta Chem. Scand.* **1989,** *43,* 471–475. doi:10.3891/acta.chem.scand.43-0471
14. Hurd, C. D.; Bonner, W. A. *J. Am. Chem. Soc.* **1945,** *67,* 1972–1977. doi:10.1021/ja01227a033
15. Helferich, B.; Günther, E.; Winkler, S. *Liebigs Ann.* **1934,** *508,* 192–205.
16. Panigot, M. J.; Curley, R. W., Jr. *J. Carbohydr. Chem.* **1994,** *13,* 293–302. doi:10.1080/07328309408009194
17. Baur, J.; Jacobson, H.; Burger, P.; Artus, G.; Berke, H.; Dahlenburg, L. *Eur. J. Inorg. Chem.* **2000,** 1411–1422. doi:10.1002/1099-0682(200007)2000:7<1411::AID-EJIC1411>3.0.CO;2-M
18. Heppert, J. A.; Boyle, T. J.; Takusagawa, F. *Organometallics* **1989,** *8,* 461–467. doi:10.1021/om00104a029
